# Gefitinib and Luteolin Cause Growth Arrest of Human Prostate Cancer PC-3 Cells via Inhibition of Cyclin G-Associated Kinase and Induction of miR-630

**DOI:** 10.1371/journal.pone.0100124

**Published:** 2014-06-27

**Authors:** Minami A. Sakurai, Yuki Ozaki, Daisuke Okuzaki, Yoko Naito, Towa Sasakura, Ayumi Okamoto, Hiroe Tabara, Takao Inoue, Man Hagiyama, Akihiko Ito, Norikazu Yabuta, Hiroshi Nojima

**Affiliations:** 1 Department of Molecular Genetics, Osaka University, Suita City, Osaka, Japan; 2 DNA-chip Development Center for Infectious Diseases, Research Institute for Microbial Diseases, Osaka University, Suita City, Osaka, Japan; 3 Department of Pathology, Faculty of Medicine, Kinki University, Osaka-Sayama, Osaka, Japan; Yokohama City University School of Medicine, Japan

## Abstract

Cyclin G-associated kinase (GAK), a key player in clathrin-mediated membrane trafficking, is overexpressed in various cancer cells. Here, we report that GAK expression is positively correlated with the Gleason score in surgical specimens from prostate cancer patients. Embryonic fibroblasts from knockout mice expressing a kinase-dead (KD) form of GAK showed constitutive hyper-phosphorylation of the epidermal growth factor receptor (EGFR). In addition to the well-known EGFR inhibitors gefitinib and erlotinib, the dietary flavonoid luteolin was a potent inhibitor of the Ser/Thr kinase activity of GAK *in vitro*. Co-administration of luteolin and gefitinib to PC-3 cells had a greater effect on cell viability than administration of either compound alone; this decrease in viability was associated with drastic down-regulation of GAK protein expression. A comprehensive microRNA array analysis revealed increased expression of miR-630 and miR-5703 following treatment of PC-3 cells with luteolin and/or gefitinib, and exogenous overexpression of miR-630 caused growth arrest of these cells. GAK appears to be essential for cell death because co-administration of gefitinib and luteolin to EGFR-deficient U2OS osteosarcoma cells also had a greater effect on cell viability than administration of either compound alone. Taken together, these findings suggest that GAK may be a new therapeutic target for prostate cancer and osteosarcoma.

## Introduction

Prostate cancer is one of the most frequently diagnosed cancers and is the leading cause of male cancer deaths worldwide [1]. Because it is driven primarily by androgen receptor (AR) signaling, advanced prostate cancer is typically treated by androgen deprivation therapy, in which surgical or medical castration is performed to block active AR signaling either by eliminating the ligand or affecting the receptor directly [2]. Although effective, androgen ablation controls metastatic prostate cancer for only a few years and almost all patients develop progressive hormone-refractory prostate cancer (HRPC). Androgen deprivation therapy is not curative for these patients even when paired with more effective therapies that target the central synthesis of testosterone, such as the CYP17A1 inhibitor abiraterone and the AR antagonist MDV3100 [3], [4]. Identification of a new target that controls AR signaling indirectly, or even a target that is unrelated to AR signaling, may be useful for developing new therapeutic approaches to HRPC.

The ubiquitously expressed cyclin G-associated kinase (GAK) regulates clathrin-mediated membrane trafficking by acting as an essential cofactor for Hsc70-dependent uncoating of clathrin-coated vesicles in the cytoplasm [5], [6]. GAK is also localized to the nucleus [7], where it acts as a transcriptional coactivator of ARs. Notably, GAK expression is increased significantly in HRPC cells [8]. Moreover, GAK plays a role in the maintenance of proper centrosome maturation and mitotic chromosome congression, and siRNA-mediated knockdown of GAK activates the spindle-assembly checkpoint, which senses protruded, misaligned, or abnormally condensed chromosomes, leading to cell cycle arrest at metaphase [9]. GAK is essential for cell growth since GAK knockout (GAK^−/−^) mice are embryonic lethal [10] and mouse embryonic fibroblast (MEF) derived from GAK^−/−^ mice fail to divide and ultimately become senescent [11] due to disruption of clathrin-mediated endocytosis. However, we previously showed that knockout mice expressing the kinase-dead form of GAK (GAK-kd) were not embryonic lethal and GAK-kd MEFs grew normally [12], suggesting that loss of GAK kinase activity may not be lethal in normal cells that express the proper amount of GAK. This evidence suggests that an inhibitor of GAK kinase activity could be used to develop a novel molecular-target therapy that selectively inhibits the growth of HRPC cells with enhanced GAK expression by controlling AR signaling indirectly.

Gefitinib [13], a tyrosine kinase inhibitor selective for the epidermal growth factor receptor (EGFR), also inhibits the kinase activity of GAK [14]. Although EGFR [15] and GAK [8] are expressed at high levels in prostate cancer and their enhanced expression is associated with poor prognosis [8], [15], administration of gefitinib alone is ineffective [16] and combined administrations of gefitinib and radiation therapy [17] or docetaxel [18] have limited therapeutic effects. Luteolin, a common dietary flavonoid found in a large variety of plants and foods [19], is another antagonist of EGFR-associated tyrosine protein kinases. Similar to gefitinib, luteolin causes growth arrest of PC-3 human prostate cancer cells via regulation of cell cycle regulatory genes [20]. Luteolin also inhibits DNA topoisomerases, the clinical targets of anticancer drugs [21]. Therefore, luteolin may be useful as an anticancer drug, although its *bona fide* target(s) and the molecular mechanisms involved in inhibition of cancer cell proliferation remain elusive due to its wide spectrum of pharmacological properties.

The aim of this study was to identify a new factor that controls AR signaling indirectly in HRPC patients, and to examine whether GAK could be targeted for the therapy of not only HRPC but also other cancers with enhanced GAK expression. Indeed, we here report that an increase in the expression of GAK in HRPC patients positively correlates with the Gleason score, a measure of the severity of the disease. Both GAK and AR localized to the nuclei of cancer cells in GAK-positive surgical specimens. An *in vitro* kinase assay revealed that luteolin and gefitinib inhibit the kinase activity of GAK with similar potency, suggesting their usefulness as inhibitors of GAK's kinase activity. Compared with the effects of either drug alone, co-administration of luteolin and gefitinib to PC-3 cells had a greater inhibitory effect on cell viability. These compounds also had a cumulative inhibitory effect on GAK protein expression that was independent of proteasome-mediated degradation. Taken together, the results presented here suggest that GAK, which is overexpressed in many cancer cells, is a novel candidate for promising targeted chemotherapy.

## Materials and Methods

### Antibodies and siRNAs

Antibodies against the following proteins were used in this study: AR (Santa Cruz Biotechnology), active caspase 3 (Cell Signaling Technology), Ki67 (DakoCytomation), Lefty (Abcam), lamin A/C (Bethyl Laboratories), EGFR (rabbit; Cell Signaling Technology), EGFR (mouse; Millipore), ERK1/2 (Cell Signaling Technology), pERK (Cell Signaling Technology), GAPDH (Fitzgerald), and α-tubulin (Sigma-Aldrich). The anti-GAK monoclonal antibodies were prepared as reported previously [7]. The Lefty1-specific siRNAs were purchased from OriGene Technologies and Gene Design.

### Chemicals and dietary supplements

The following chemicals and dietary supplements were used in this study: gefitinib (Tocris Bioscience), erlotinib (Kemprotec), SB203580 (LC Laboratories), LutiMax (CalComp Nutrition), Oryza luteolin (Oryza Oil & Fat Chemical), luteolin (Sigma-Aldrich), resveratrol (Sigma-Aldrich), and DMSO (Sigma-Aldrich).

### Cell culture

The PC-3 cells were provided by the Japanese Cancer Research Resources Bank. All other human cancer cells were purchased from the American Type Culture Collection. The cells were maintained in 5% CO_2_ at 37°C in Dulbecco's modified Eagle's medium supplemented with 10% fetal bovine serum (FBS, Hyclone Laboratories), 100 U/ml penicillin, and 100 µg/ml streptomycin. WT (GAK-kd^+/+^) and GAK-kd^−/−^ MEFs were maintained in MEF medium (Dulbecco's modified Eagle's medium supplemented with 10% FBS, penicillin, streptomycin, and 50 mM 2-mercaptoethanol) as described previously [22].

### EGF stimulation

After two washes in phosphate-buffered saline (PBS), MEFs were cultured in low-serum MEF medium (containing 0.5% FBS) for 12 h. Mouse EGF (Sigma) was added to the culture medium at a final concentration of 10 ng/ml (for western blot analysis) or 100 ng/ml (for immunofluorescence), and the cells were then incubated in 5% CO_2_ at 37°C for the indicated times.

### FACS analysis

Cells were stained using the Cycletest Plus DNA Reagent Kit (BD Bioscience), according to the manufacturer's instructions. Analysis was performed using a FACS Calibur instrument with CellQuest software (BD Bioscience).

### Growth curve analysis

Approximately 1.0×10^3^ PC-3 cells were seeded into a 3.5 cm Petri dish and incubated at 37°C overnight. Gefitinib, luteolin, and resveratrol were then dissolved in DMSO and added to the culture medium at time zero.

### Cell viability measurement using miR-630

Expression of miR-630 from the miRNASelect pEP-hsa-mir-630 expression vector was performed according to the manufacturer's instructions (Cell Biolabs). To determine viability, the cells were plated into 6-well plates at a density of 1×10^5^ cells per well and then trypsinized at the indicated time-points. The numbers of proliferating cell were determined using a Countess Automated Cell Counter (Invitrogen).

### Histological analysis

Surgical specimens from patients undergoing radical prostatectomy were fixed in 10% buffered formalin, embedded in paraffin, and then cut into 4 µm thick serial sections. The first sections were stained with hematoxylin and eosin and used for pathological diagnosis of the inflamed region. The remaining three sections were subjected to immunohistochemical analyses, as described previously [23]. Briefly, deparaffinized sections were autoclaved in 0.1 M citrate buffer, blocked with bovine serum albumin, and then incubated with primary antibodies in PBS containing 2% bovine serum albumin. Secondary antibody incubations and signal enhancement reactions were performed using the Histofine Simple Stain kit (Nichirei) and the color was developed using aminoethlcarbazole (Impact AEC; Vector Laboratories). The sections were counterstained with hematoxylin for nuclear visualization and then mounted using Ultramount Aqueous Permanent Mounting Medium (Dako). Images were recorded using a microscope (BX51; Olympus) equipped with a CCD camera (DP72; Olympus).

### Western blot analysis

To prepare whole cell lysates, the cells were lysed at 4°C for 30 min in modified RIPA buffer (10 mM Tris-HCl pH 7.5, 150 mM NaCl, 1 mM EDTA, 1% NP-40, 1 mM dithiothreitol, 0.1% SDS, and 0.1% deoxycholate) supplemented with 1% protease inhibitor cocktail (Sigma-Aldrich), 1 mM NaF, 1 mM Na_3_VO_4_, and 10 mM β-glycerophosphate. After centrifugation, the cleared lysates were subjected to SDS-PAGE. The resolved proteins were transferred to PVDF membranes, which were blocked and then immunoblotted with the indicated antibodies in Tris-buffered saline and Tween 20 containing 5% nonfat milk. The immunoreactive protein bands were visualized using Western Lightning Plus ECL reagents (PerkinElmer).

### Purification of recombinant proteins

Purification of GST-tagged proteins for kinase assays was performed as described previously [22]. Briefly, *E. coli* BL21 cells transformed with the pGEX plasmid were incubated in Luria-Bertani broth at 37°C until they reached an absorbance of 0.4–0.8 at 600 nm. Expression of each recombinant protein was induced by overnight exposure of the cells to 0.5 mM IPTG at 20°C. The cells were then collected and lysed by sonicating in PBS containing 1% Triton X-100, 1 µg/ml leupeptin, 1 µg/ml aprotinin, 1 µg/ml pepstatin A, 1 mM benzamidine, 100 µg/ml phenylmethylsulfonyl fluoride, 1 mM NaF, and 1 mM Na_3_VO_4_. After centrifugation, the cleared lysate was adsorbed to Glutathione Sepharose 4B (Amersham Pharmacia Biotech), rinsed with PBS, and then eluted with a buffer containing 10 mM reduced glutathione (Nacalai Tesque).

### *In vitro* kinase assay of GAK

*In vitro* kinase assays were performed using GST-tagged human AR (∼150 µg/ml) as the test sample and purified GST-tagged human GAK (∼100 µg/ml) as the test kinase. A purified fragment of the B′γ3 subtype of mouse PP2A (B′γ; ∼10 µg/ml) was used as a positive control GAK substrate, as described previously [22]. Aurora A kinase (∼50 µg/ml) and Lats1/2 kinase (∼25 µg/ml; in the presence or absence of Mob1A (∼50 µg/ml) as its activator) were used as positive control enzymes, and a kinase-dead (KD) form of Aurora A kinase (∼50 µg/ml) was used as a negative control for phosphorylation of the AR. The kinases and substrate proteins were incubated for 30 min at 30°C in 10 µl reaction buffer (10 mM HEPES pH 7.5, 50 mM NaCl, 10 mM MgCl_2_, 5 mM MnCl_2_, 1 mM DTT, 5 mM NaF, and 50 mM β-glycerophosphate) containing 5 µM ATP and 10 µCi [γ-^32^P] ATP (PerkinElmer). Where required, gefitinib, erlotinib, SB203580, resveratrol, and luteolin were included at the indicated concentrations. The reaction was stopped by the addition of 4× Laemmli buffer (0.4 M Tris-Cl pH 6.8, 8% SDS, 20% glycerol, 10% 2-mercaptoethanol, and 0.2% bromophenol blue). The samples were resolved by gradient SDS-PAGE using a Multigel II Mini apparatus (Daiichi Pure Chemicals) and were detected by autoradiography. The amounts of proteins loaded onto the gel were monitored by staining with Coomassie Brilliant Blue (Bio-Rad) or SimplyBlue™ SafeStain (Life Technologies).

### Human genome microarray analysis

Microarray analyses were performed as single-color hybridizations using Agilent Whole Human Genome Oligonucleotide Microarrays (4×44K; Product code: 4112F). Total RNA was extracted from PC-3 cells 24 h after treatment with DMSO, 60 µM luteolin, 60 µM gefitinib, or 60 µM luteolin plus 60 µM gefitinib using an miRNeasy Mini kit according to the manufacturer's instructions (Qiagen). The quality of the RNA was determined using the RNA 6000 Nano LabChip Kit on the Agilent 2100 Bioanalyzer (Agilent Technologies). The RNA was reverse-transcribed using AffinityScript reverse transcriptase (Agilent Technologies) and oligo-dT primers containing the T7 RNA polymerase promoter sequence. *In vitro* transcription was then performed using T7 RNA polymerase and a Low input Quick-Amp Labeling Kit (Agilent Technologies) to label the cRNAs with Cy3-CTP (Amersham Pharmacia Biotech, Piscataway, NJ). Purified Cy3-labeled cRNAs (1650 ng) were hybridized to the microarrays. Washing, scanning, and gene analyses were performed according to the manufacturer's protocol (Agilent Technologies). Agilent Feature Extraction software (v. 10.5.1) was used to assess spot quality and extract feature intensity statistics. The Subio Platform and Subio Basic Plug-in (v1.15; Subio Inc.) were then used to calculate between-sample fold-changes, which were analyzed by one-sample Student's *t*-tests. The microarray data have been deposited in the Gene Expression Omnibus database (www.ncbi.nlm.nih.gov/geo) under accession number GSE53180.

### Human miRNA microarray analysis

The miRNA microarray analysis was performed using Agilent SurePrint Human v18.0 miRNA arrays, which contain 20–40 features targeting 1919 human miRNAs cataloged in version 8.0 of the Sanger database (design ID 025416). Aliquots of total RNA (100 ng) were used to prepare miRNA probes, according to the Agilent protocol (v. 2.4). Briefly, total RNA was dephosphorylated with calf intestine alkaline phosphatase, denatured with DMSO, and then labeled with Cyanine 3-pCp using T4 RNA ligase and the miRNA Labeling Kit. The probes were hybridized to the arrays for 20 h at 55°C with rotation using the Agilent miRNA Hybridization Kit. Duplicate microarrays were performed for PC-3 cells treated with DMSO, luteolin, gefitinib, or luteolin and gefitinib. The slides were washed with Gene Expression Wash Buffer 1 (Agilent) at room temperature for 5 min and then with Gene Expression Wash Buffer 2 (Agilent) at 37°C for a further 5 min. After hybridization and washing, the slides were scanned using an Agilent scanner (G2505C). Images were extracted using Agilent Feature Extraction software (v. 10.7.3) and Agilent GeneSpring GX software (v. 12.6). The total gene signal from GeneView data files (extracted with default settings in Agilent Feature Extraction software) was used. Differences in miRNA expression levels were normalized using the PC-3 treated DMSO sample as a control. aFold-changes greater than 2.0 and *P*-values less than 0.05 (Student's *t*-test) were considered for further analysis. The miRNA microarray data have been deposited in the Gene Expression Omnibus database under accession number GSE53178.

### Quantitative real-time PCR

Quantitative RT-PCR analyses were performed using an ABI PRISM 7900 instrument (PE Applied Biosystems) and Assay-on-Demand TaqMan probes for Lefty1 (Hs00764128_s1), miR-630 (hsa-mir-630), and miR-5703 (hsa-mir-5703), according to the manufacturer's protocols (Agilent Technologies).

### Statistical analysis

Error bars for all data represent SDs from the mean. *P*-values were calculated using Student's *t*-tests, unless otherwise stated.

### Ethics

Human samples were collected by biopsy from the prostates of cancer patients with relevant clinical data at the Kinki University Hospital. All experiments using human samples were approved by the ethics committee of Osaka University (Approval #326) and Kinki University (Approval #23-088); our ethics committee waived the need for consent. Surgical specimens from patients undergoing radical prostatectomy at Kinki University Hospital (Osaka, Japan) were fixed in 10% buffered formalin, embedded in paraffin, and then cut into 4 µm thick serial sections.

## Results

### GAK is localized mainly to the nucleus in cancer cells

**Figure 1:**
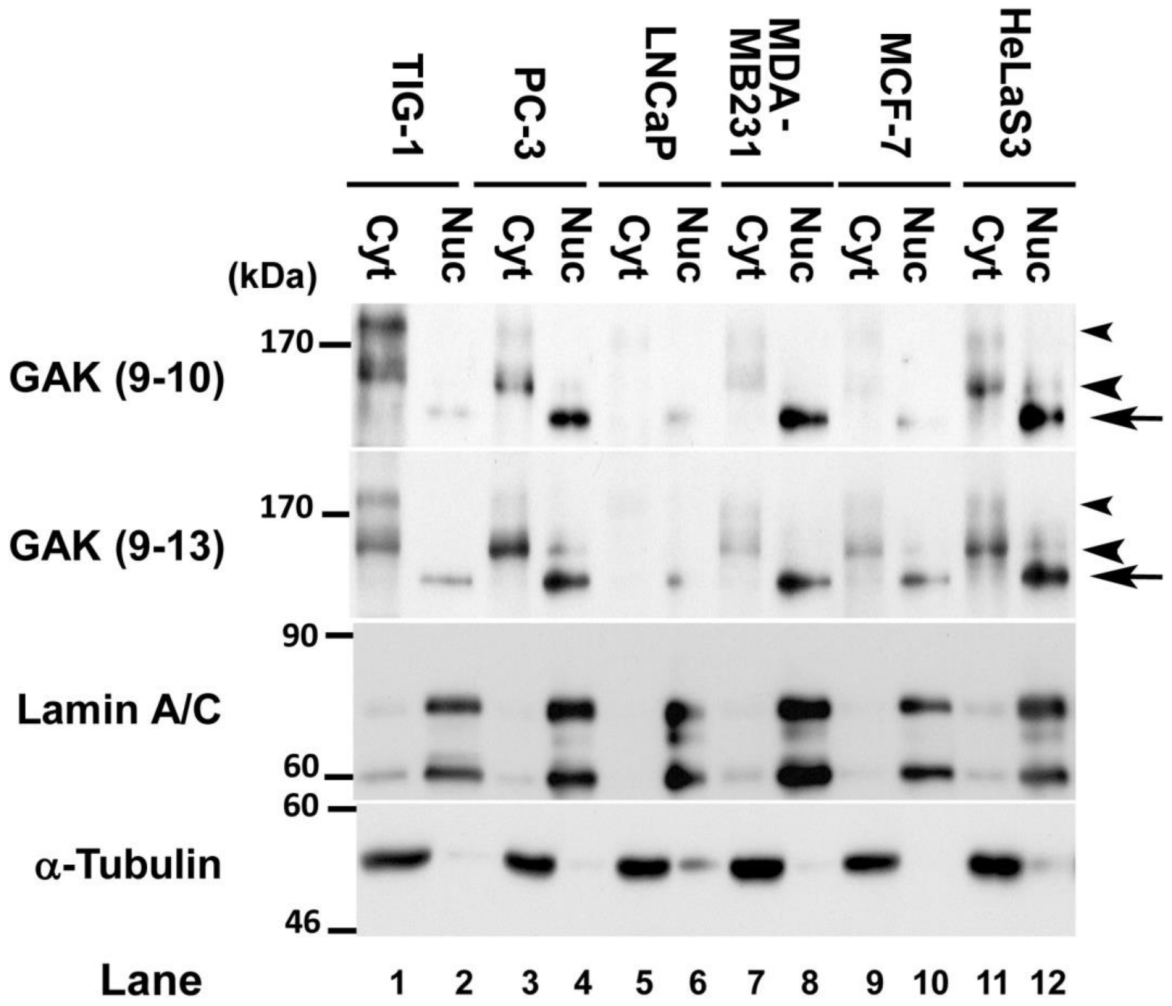
GAK is overexpressed in the nuclei of cancer cells. Western blot analysis of GAK, lamin A/C, and α-tubulin (control) in cytoplasmic (Cyt) and nuclear (Nuc) extracts from prostate cancer (PC-3 and LNCaP), breast cancer (MDA-MB231 and MCF-7) and cervical cancer (HeLaS3) cell lines. Lamin A/C was used as a nuclear marker. Two different anti-GAK antibodies were used. The arrows and arrowheads indicate the GAK bands identified primarily in the nuclear and cytoplasmic fractions, respectively.

Although GAK localizes mainly to the cytoplasm in normal cells such as TIG-1, we recently reported that a fraction of the protein is also found in the nucleus [7]. Notably, a previous study using a neo-adjuvant hormone therapy tissue microarray demonstrated that GAK expression increases significantly during the progression of prostate cancer to androgen independence [8]. To determine if GAK expression is augmented in the nuclei of cancer cells, immunoblots were used to measure GAK protein levels in cytoplasmic and nuclear fractions of various cancer cell lines. In hormone-insensitive human prostate cancer cells (PC-3), GAK was expressed at a higher level in the nucleus than in the cytoplasm; this result was confirmed using two different monoclonal antibodies (lanes 3 and 4 in Fig. 1). Although the amount of GAK was low, the nuclei of hormone-sensitive human prostate cancer cells (LNCaP) also expressed GAK (lane 6 in Fig. 1). Moreover, the nuclear GAK band migrated faster than the cytoplasmic GAK band, suggesting distinct modifications of the protein in these cellular compartments. Similar results were also observed for MDA-MB231 and MCF-7 breast cancer cells, as well as HeLaS3 cervical cancer cells (Fig. 1). By contrast, GAK was present at higher levels in the cytoplasmic fraction than the nuclear fraction of TIG-1 normal fibroblasts (Fig. 1). Notably, hormone-insensitive cancer cells (PC-3 and MDA-MB231) expressed GAK at higher levels than hormone-sensitive cancer cells (LNCaP and MCF-7). Taken together, these results indicate that GAK is modified and overexpressed in cancer cells, especially metastatic cells.

### Nuclear GAK is expressed at high levels in surgical specimens from prostate cancer patients

**Figure 2:**
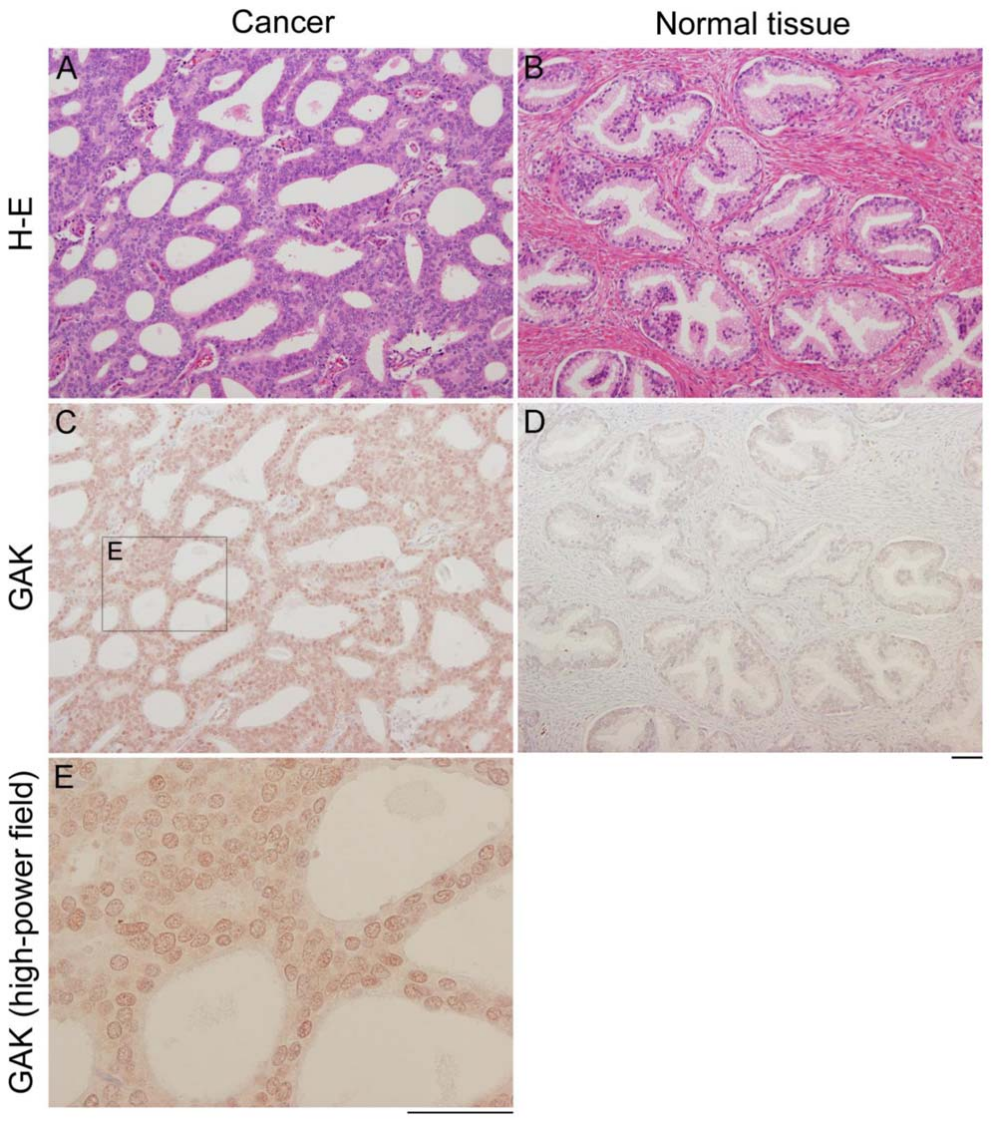
GAK is overexpressed in radical prostatectomy specimens from prostate cancer patients. A–D, hematoxylin and eosin (H-E) staining (A, B) and anti-GAK immunostaining (C, D) of representative radical prostatectomy sections of cancerous (A, C) and normal (B, D) tissues from prostate cancer patients (n = 42). Scale bar = 50 µm. E, higher power magnification of the boxed area shown in C. Scale bar = 50 µm.

To determine if nuclear overexpression of GAK occurs *in vivo*, immunohistochemical analyses of normal and cancerous surgical specimens from 42 prostate cancer patients were performed. Although GAK was only expressed weakly in the normal tissues, the protein was expressed at high levels in the nuclei of cancerous cells (Fig. 2A–E). A chi-squared test revealed a statistically significant positive correlation between GAK immunostaining and the Gleason score (Table 1 and Table S1).

**Table 1:** Statistical analysis of the relationship between the Gleason score and GAK immunostaining in the prostatectomy specimens.

Gleason	Immunostaining of GAK		Total
Score	Positive (%)	Negative (%)	
≦7	4 (12.1)	29 (87.9)	33
8 ≦	5 (55.6)	4 (44.4)	9
Total	9	33	42

*P*<0.01.

Over-activation of AR signaling is associated with progression of androgen-dependent prostate-cancers. Because GAK interacts with the AR *in vitro* [8], immunostaining was used to determine if GAK also colocalizes with the AR *in vivo*. Nuclear localization of GAK and the AR was detected in the nuclei of cancer cells from all GAK-positive surgical specimens (n = 4 patients with a Gleason score ≤7 and n = 5 patients with a Gleason score ≥8) (Fig. S1). However, despite their nuclear localization, direct phosphorylation of the AR by GAK was not detected by an *in vitro* kinase assay (Fig. S1E). Moreover, in three independent patient samples, GAK and the AR did not colocalize with Ki-67, a cancer antigen that is found primarily in growing cells and is absent in resting cells (Fig. S2). This finding suggests that colocalization of GAK and the AR does not necessarily correlate with the growth of cancer cells.

### Cytoplasmic GAK regulates EGFR-mediated signaling pathways

**Figure 3:**
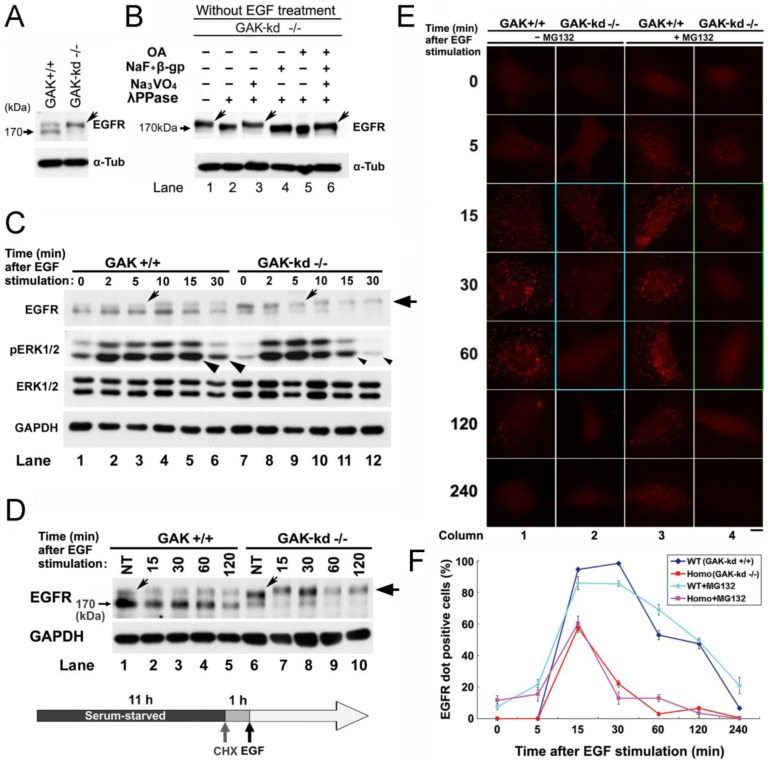
Loss of GAK activity leads to constitutive hyper-phosphorylation of the EGFR. A, western blot analysis of EGFR expression in WT (GAK-kd^+/+^) and mutant (GAK-kd^−/−^) MEFs. B, the effects of a Tyr-phosphatase inhibitor (50 mM Na_3_VO_4_) and Ser/Thr phosphatase inhibitors (50 mM NaF and 50 mM β-glycerophosphate, or 2.5 µM okadaic acid) on the inhibition of EGFR phosphorylation by λ-phosphatase (λPPase; 200 U). A, B, the arrows indicate the phosphorylated EGFR protein. Alpha-tubulin (α-Tub) was used as a loading control. (C, D) Western blot analysis of expression levels of the EGFR and ERK1/2 in WT (+/+) and GAK-kd (−/−) MEFs following EGF stimulation for the indicated times. Cycloheximide (50 µg/ml) was added to the culture medium 1 h prior to EGF (10 µg/ml) to inhibit novel protein synthesis. The tilted and horizontal arrows indicate the phosphorylated and hyper-phosphorylated EGFR bands, respectively. GAPDH was used as a loading control. C, the arrowheads indicate differential expression of phosphorylated ERK1/2 in WT and GAK-kd MEFs. D, NT, non-treated. E, immunostaining of the EGFR protein in WT (+/+) and mutant (−/−) MEFs treated with or without the proteasome inhibitor MG132 (50 µg/ml). Notable panels are encircled by turquoise and green lines. F, the numbers of EGFR-positive cells in WT and GAK-kd (Homo) cells in the presence or absence of MG132. The data are represented as the mean ± SEM of n = 3 independent experiments at each time point.

Down-regulation of GAK reduces the tyrosine kinase activity of the EGFR and alters its downstream signaling pathways [5]. To determine if this regulation is mediated by the Ser/Thr kinase activity of GAK, we investigated the behavior of the EGFR in GAK-kd MEFs [12]. An immunoblot analysis of wild-type (WT) and GAK-kd MEFs revealed the presence of two EGFR proteins in both samples, the larger of which was predominant in GAK-kd MEFs (Fig. 3A). The larger band disappeared after treatment of the cells with λ-phosphatase (Fig. 3B, lane 2), suggesting that it represented a phosphorylated form of the EGFR. The Tyr-phosphatase inhibitor Na_3_VO_4_, but not the Ser/Thr phosphatase inhibitors NaF, β-glycerophosphate, and okadaic acid, abolished this effect of λ-phosphatase, suggesting that Tyr residues in the EGFR are constitutively phosphorylated in GAK-kd MEFs in the absence of EGF stimulus (Fig. 3B). Similarly, when the MEFs were serum starved for 11 h, treated with cycloheximide for 1 h to inhibit novel protein synthesis, and then stimulated by the addition of EGF, phosphorylation of the EGFR was more prominent in GAK-kd MEFs than WT MEFs (Fig. 3C and 3D). Notably, an even larger EGFR protein was detected in both WT and GAK-kd MEFs 10 min after EGF stimulation (Fig. 3C and 3D), suggesting that EGF increased the number of phosphorylated Tyr residues in the EGFR. This hyper-phosphorylation accelerated the dephosphorylation of phosphorylated extracellular signal-regulated kinases 1/2 (pERK1/2) (Fig. 3C), which are downstream effectors of the EGFR, suggesting premature diminution of EGFR signaling.

Immunostaining showed that the levels of EGFR in the cytoplasm of GAK-kd MEFs were lower than those in the cytoplasm of WT MEFs, suggesting abnormal internalization of the EGFR in the knockout cells (column 2 panels of Fig. 3E). Similar results were obtained when the experiment was repeated in the presence of the proteasome inhibitor MG132, suggesting that the abnormality was not due to the degradation of EGFR proteins (column 4 panels of Fig. 3E). Moreover, the number of EGFR-positive cells was lower in GAK-kd MEFs than WT MEFs, and this phenotype was also independent of proteasome inhibition (Fig. 3F). These results suggest that constitutive hyper-phosphorylation of the EGFR caused by a lack of GAK activity hampers proper localization of the receptor, leading to reduced activation of EGFR-mediated growth signaling. The results described above are consistent with a recent report that MEFs from GAK conditional knockout mice and GAK knockdown cells prepared using small hairpin RNAs display altered intracellular EGFR trafficking, undergo growth arrest, and have reduced pERK1/2 signaling [11]. Taken together, these data suggest that a lack of GAK activity disturbs proper activation of EGFR-mediated signaling pathways.

### Luteolin and gefitinib inhibit the kinase activity of GAK

**Figure 4:**
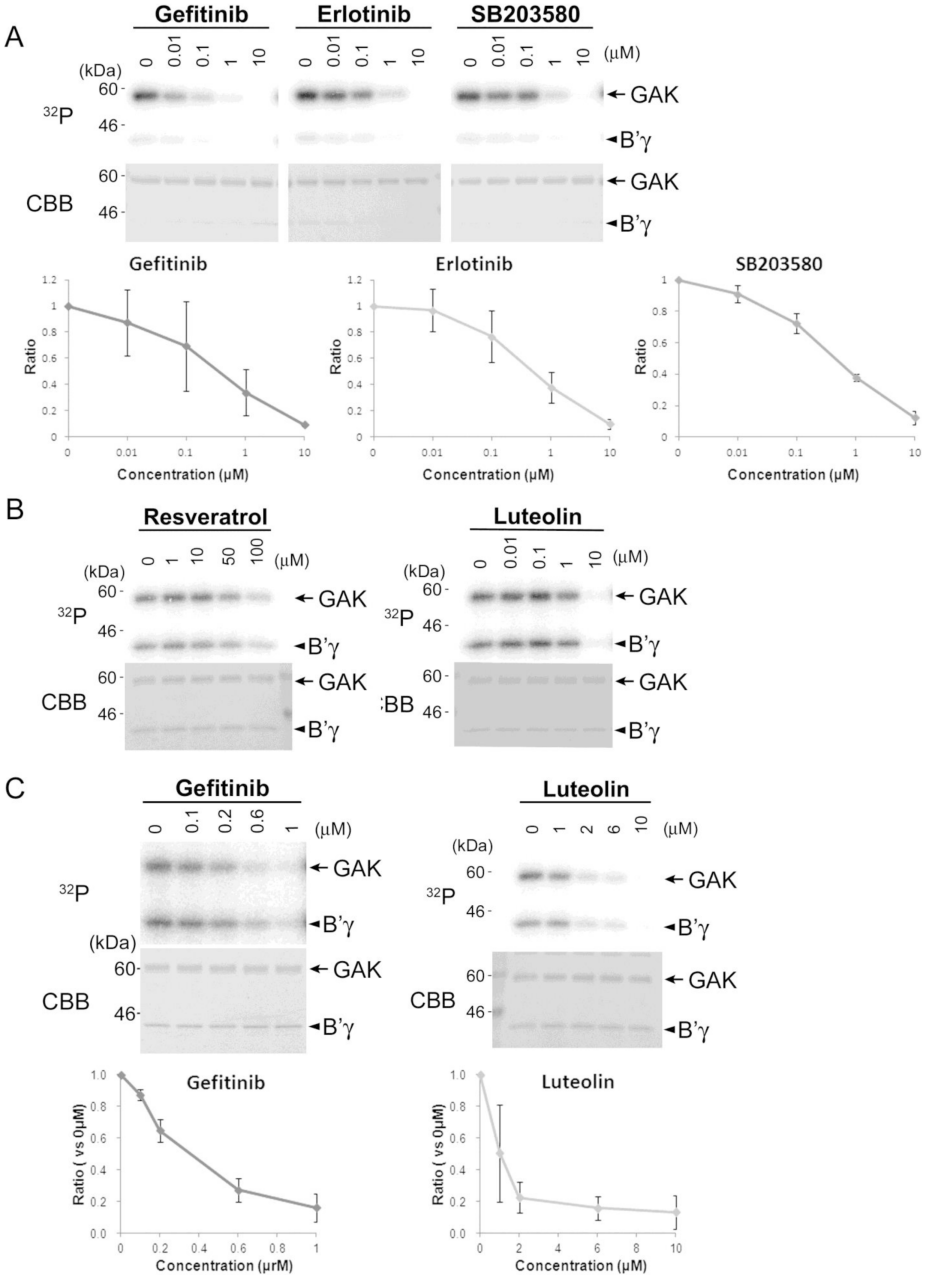
Luteolin and erlotinib inhibit GAK activity similarly to gefitinib and SB203580. A–C, representative SDS-PAGE analysis of proteins subjected to *in vitro* kinase assays using ^32^P-γATP, GAK (∼100 µg/ml) as the enzyme, PP2A B′γ (∼10 µg/ml) as the substrate, and the indicated concentrations of gefitinib, erlotinib, and SB203580 as inhibitors. The signals incorporated were detected by autoradiography and the amounts of proteins loaded were assessed by Coomassie Brilliant Blue (CBB) staining. The graphs show the intensity ratios of phosphorylated PP2A B′γ and GAK relative to that of non-treated samples. The data are represented as the mean ± SEM of n = 3 independent experiments at each concentration.

Gefitinib inhibits GAK, the EGFR, and receptor-interacting serine/threonine kinase *in vitro* [14]. To determine whether gefitinib inhibits GAK activity directly, an *in vitro* kinase assay was performed using PP2A B′γ, a known target of GAK, as a substrate [22]. Gefitinib inhibited GAK autophosphorylation and GAK-mediated phosphorylation of PP2A B′γ in a dose-dependent manner, suggesting that gefitinib inhibits GAK kinase activity directly (Fig. 4A). The effects of other kinase inhibitors, such as the well-known EGFR inhibitor erlotinib [24] and the p38α and GAK inhibitor SB203580 [25], on GAK activity were also examined. Both of these compounds inhibited GAK activity similarly, although they were slightly less potent than gefitinib (Fig. 4A). *In vitro* kinase assays were also used to determine the effects of luteolin (a flavonoid) and resveratrol (a polyphenol) on GAK activity; these compounds inhibit the growth of prostate cancer cell lines [20], [26]. Notably, 10 µM luteolin inhibited GAK activity drastically, whereas the same concentration of resveratrol had only a slight inhibitory effect (Fig. 4B). A further analysis revealed that 2 µM of luteolin was required to inhibit GAK activity (Fig. 4C). These results indicate that, in addition to gefitinib, luteolin and erlotinib are novel inhibitors of GAK.

### Co-administration of gefitinib and luteolin causes death of PC-3 cells

**Figure 5:**
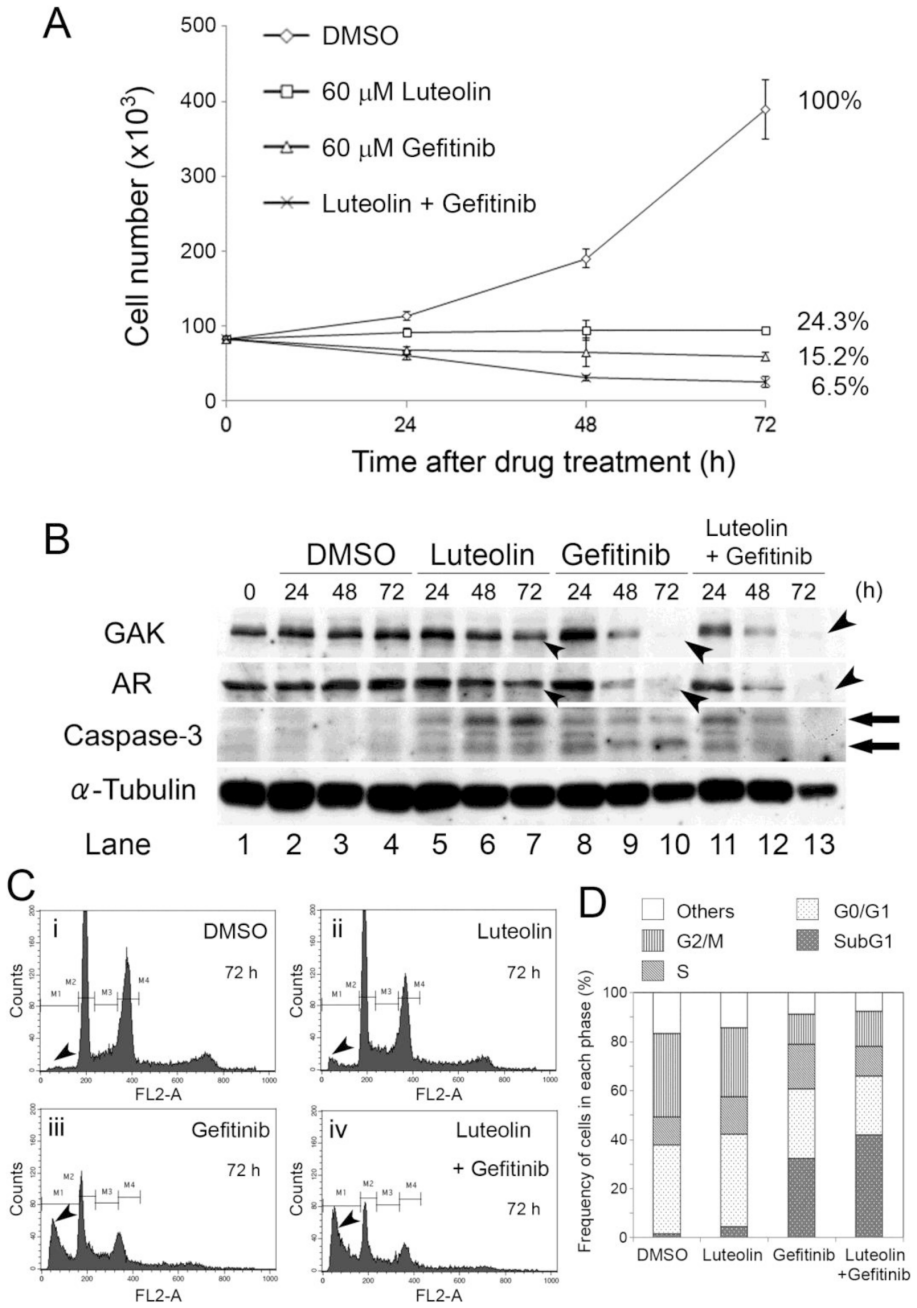
Luteolin and gefitinib induce death of PC-3 cells. A, the numbers of viable PC-3 cells 0, 24, 48, and 72 h after the addition of solvent alone (DMSO), 60 µM luteolin, 60 µM gefitinib, or 60 µM luteolin plus 60 µM gefitinib to the culture medium. The data are represented as the mean ± SEM of n = 3 independent experiments at each concentration. B, immunoblot analyses of GAK, AR, caspase-3, and α-tubulin (control) protein levels in PC-3 cells exposed to solvent alone (DMSO), 60 µM luteolin, 60 µM gefitinib, or 60 µM luteolin plus 60 µM gefitinib for 0–72 h. The arrowheads indicate reduced expression levels of GAK and AR in cells exposed to the drugs for 72 h. The arrows indicate the emergence of activated caspase-3, which suggested that gefitinib and luteolin induced apoptosis. C, FACS analysis of PC-3 cells PC-3 cells exposed to solvent alone (DMSO), 60 µM luteolin, 60 µM gefitinib, or 60 µM luteolin plus 60 µM gefitinib for 72 h. The arrowheads indicate the subG1 populations. D, the percentages of PC-3 cells in the indicated phases of the cell cycle after exposure to the same treatments for 72 h.

Next, we examined the effect of co-administration of gefitinib and luteolin on the growth of PC-3 prostate cancer cells. We first used genomic DNA sequencing to examine for the presence of mutations in the EGFR gene in PC-3 cells that were reported previously to be harbored frequently by lung cancer cells [27]. As shown in Fig. S3, we found no mutations at such sites in the genome of PC-3, which suggests that a low concentration of gefitinib (∼1 µM) may not be effective in PC-3 cells. Indeed, a higher concentration of gefitinib (60 µM) was required to arrest the growth of PC-3 cells. Interestingly, when the numbers of proliferating cells were counted after exposure of the cells to 60 µM luteolin, 60 µM gefitinib, or a combination of these drugs for 24, 48, or 72 h, we found a cumulative inhibitory effect of gefitinib and luteolin on cell growth (Fig. 5A). Control cells were treated with dimethylsulfoxide (DMSO) alone. The percentages of surviving cells 72 h after exposure to luteolin alone, gefitinib alone, or luteolin plus gefitinib were 24.3%, 15.2%, and 6.5%, respectively.

Reductions in AR protein levels were observed after exposure of the cells to gefitinib alone or gefitinib plus luteolin for 48 h, and AR protein levels were almost undetectable after 72 h exposure to these drugs (Fig. 5B). By contrast, administration of luteolin alone caused only a modest decrease in the AR protein level (Fig. 5B). The expression levels of activated/cleaved caspase-3 proteins were higher in drug-treated cells than DMSO-treated cells, suggesting the induction of apoptosis by luteolin and gefitinib (Fig. 5B). A fluorescence-activated cell sorting (FACS) analysis revealed the appearance of subG1 populations following exposure of the cells to luteolin, gefitinib, or a combination of these drugs for 72 h (Fig. 5C). The percentages of subG1 cells in gefitinib-treated and dual-treated populations were 32.5% and 41.9%, respectively (Fig. 5D). By contrast, the percentage of subG1 populations in luteolin-treated cells (4.5%) was similar to that in control cells (1.6%). These results suggest that gefitinib and luteolin induce apoptosis of PC-3 cells, although luteolin is less potent than gefitinib. Additional studies in which PC-3 cells were exposed to various concentrations of gefitinib, luteolin, and erlotinib showed that the effects of erlotinib on cell growth are similar to that of gefitinib (Fig. S3A). Furthermore, the effect of a combination of gefitinib and luteolin on cell growth was similar to that of a combination of erlotinib and luteolin (Fig. S3A).

Resveratrol inhibits the growth of several prostate cancer cell lines [28]; however, the inhibitory effect of a combination of 60 µM luteolin and 60 µM gefitinib on growth of PC-3 cells was not augmented by addition of the same concentration of resveratrol (Fig. S3B-i). Although a FACS analysis revealed that treatment of PC-3 cells with resveratrol caused cell cycle arrest at the S phase, resveratrol had no other effects on the cell cycle pattern of cells exposed to luteolin and gefitinib (Fig. S3-ii, iii).

Because luteolin can be administered as a dietary supplement, we determined the effects of commercially available luteolin tablets (LutiMax, CalComp Nutrition Inc.; and Oryza Oil & Fat Chemical Co., Ltd) on PC-3 cell growth. Based on the luteolin concentrations listed by the manufacturers, the inhibitory effects of these dietary supplements on cell growth were similar to those of the pure compound (Fig. S3C). Therefore, luteolin dietary supplements may also be useful as therapeutic agents at the bedside.

### A number of genes are up-regulated following exposure of PC-3 cells to gefitinib and luteolin

Next, a DNA microarray analysis was performed to investigate the molecular mechanisms involved in the induction of cell death by luteolin and gefitinib. To avoid confounding results due to the secondary effects of apoptosis on gene expression, PC-3 cells were examined after a 24 h exposure to 60 µM luteolin and/or 60 µM gefitinib; this time point was chosen because the subG1 population had not yet appeared (Fig. S3D). Fold-changes in gene expression were determined by comparing the hybridization signal intensities at time zero (before the addition of drugs to PC-3 cells) to those at 24 h after the addition of DMSO, luteolin, gefitinib, or luteolin plus gefitinib. The expression levels of more than 30 genes were up-regulated by luteolin alone, gefitinib alone, and a combination of these drugs (Fig. S4A).

Notably, left-right determination factor 1 (Lefty1), an inhibitor of the embryonic Nodal-signaling pathway whose reactivation is associated with tumor progression and the growth of prostate cancer cells [29], but not Lefty2, was up-regulated conspicuously (77.50-fold) following exposure of the cells to luteolin (Table S2 and Fig. S4A). Lefty1 was also up-regulated to a much smaller extent by gefitinib (5.12-fold) and luteolin plus gefitinib (9.90-fold). The stimulatory effects of luteolin alone and gefitinib alone on Lefty1 mRNA expression were confirmed by duplicate quantitative real-time polymerase chain reactions (qRT-PCRs); however, these experiments did not confirm the stimulatory effect of the combination of these two drugs on Lefty1 mRNA expression (Fig. S4B-i). Western blot analyses showed that the Lefty1 protein levels were increased slightly 24 and 48 h after exposure of the cells to luteolin alone, gefitinib alone, or luteolin plus gefitinib, but these levels declined after 72 h (Fig. S4B-ii).

The commercially available anti-Lefty1 antibody used here recognized both Lefty1 and Lefty2, because the amino acid sequences of these two proteins share 96% identity [30]. However, in the miRNA array analysis, the Lefty2 mRNA level remained very low after luteolin and/or gefitinib administration (Fig. S4A); therefore, we surmise that the contribution of Lefty2 to the band intensities of the immunoblots was negligible. To confirm this hypothesis, we used siRNAs to knock down Lefty1 mRNA and showed that the 41 kDa protein band observed in the immunoblots was down-regulated (Fig. S4B-iii). Lack of induction of Lefty1 protein expression by luteolin was also confirmed by this immunoblotting (Fig. S4B-iii). Taken together, these data indicate that Lefty1 protein levels were not enhanced markedly by treatment of PC-3 cells with gefitinib and/or luteolin. Therefore, we conclude that Lefty1 is not involved in the inhibitory effects of these drugs on PC-3 cell growth. The other, less significantly up-regulated genes, identified by the microarray were not examined further because they are not directly related to growth inhibition or apoptosis of PC-3 cells.

### Gefitinib and luteolin induce miR-630 expression in PC-3 cells

Notably, GAK protein expression was reduced dramatically following exposure of PC-3 cells to luteolin and/or gefitinib for 72 h (Fig. 5B and S4B-ii). This decrease in protein expression was not due to proteasome-mediated degradation of GAK because it was not affected by addition of the proteasome inhibitor GM132 (Fig. S4B-iii). This result suggests that synthesis of the GAK protein is inhibited by luteolin, which may be due to miRNA-mediated translational inhibition. Moreover, treatment of PC-3 cells with luteolin produced faster migrating GAK protein bands in immunoblots (Fig. S4B-iii), suggesting that GAK is modified in luteolin-exposed cells.

**Figure 6:**
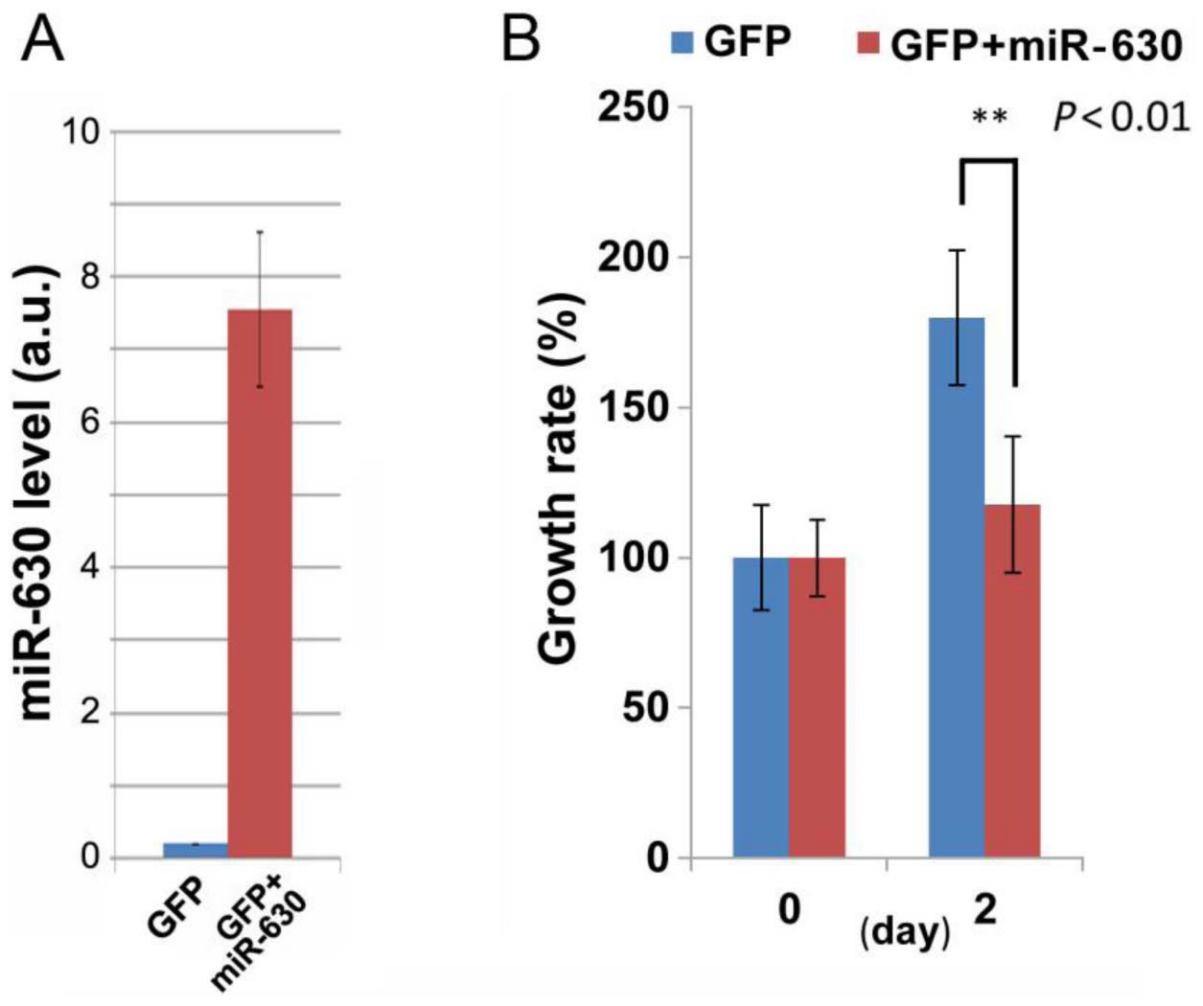
Exogenous expression of miR-630 inhibits the growth of PC-3 cells. A, confirmation by qRT-PCR of the successful overexpression of exogenous miR-630 from the pEP-hsa-miR-630 expression vector in PC-3 cells. The data are represented as the mean ± SEM of n = 3 independent experiments. B, The growth rate of PC-3 cells transfected with the pEP-hsa-miR-630 expression vector. Cells were also transfected with GFP to monitor the successful expression of miR-630. The data are represented as the mean ± SEM of n = 3 independent experiments for each condition. *P*<0.01 by a Student's *t*-test.

To determine if miRNAs are involved in the growth inhibitory and cell death inducing effects of luteolin and gefitinib, miRNA array analyses were performed using RNA from PC-3 cells treated with 60 µM luteolin and/or 60 µM gefitinib for 24 h. The expression levels of miR-630 and miR-5703 in gefitinib-treated cells were approximately 6.2-fold and 5.5-fold higher, respectively, than those in DMSO-treated cells. By contrast, luteolin increased the levels of these miRNAs by only 1.4-fold (Table S3). The effect of overexpression of exogenous miR-630 on PC-3 cell growth was determined using a commercially available expression vector (pEP-hsa-miR-630). Successful exogenous expression was confirmed by qRT-PCR (Fig. 6A) and co-transfection with a GFP vector. PC-3 cell growth was inhibited significantly by stable expression of exogenous miR-630 (Fig. 6B). These results suggest that luteolin and gefitinib induce miR-630 expression, leading to growth arrest of PC-3 cells.

### Co-administration of gefitinib and luteolin inhibits the growth of osteosarcoma cells

GAK is overexpressed in U2OS osteosarcoma cells, which lack EGFR expression, and knockdown of GAK inhibits their proliferation [31]. Therefore, we hypothesized that luteolin and/or gefitinib may induce apoptotic cell death of U2OS cells as well as PC-3 cells. In the initial experiments, we were unable to determine the combined effects of 60 µM luteolin and 60 µM gefitinib on cell viability because this concentration of gefitinib was already very toxic to U2OS cells 24 h after addition (Fig. S5A). Therefore, in subsequent experiments, the concentration of gefitinib was reduced to 20 µM because a previous report indicated that this concentration does not affect U2OS cell viability [32]. U2OS cells were exposed to 60 µM luteolin and/or 20 µM gefitinib for 24, 48, or 72 h. At the 72 h time point, the percentage of viable cells in the group exposed to both drugs was 1.1%, while those in the groups exposed to luteolin alone and gefitinib alone were 8.5% and 42.0%, respectively (Fig. S5B). Since luteolin is readily available as a dietary supplement (LutiMax) that can be administered at the bedside, the combined use of LutiMax and gefitinib may be an effective therapeutic option for osteosarcoma patients.

## Discussion

This study shows that GAK could be used as a target for not only the therapy of HRPC but also other cancers with enhanced GAK expression. Indeed, GAK is modified and overexpressed in cancer cell lines (Fig. 1) and that GAK is overexpressed in surgical specimens from prostate cancer patients (Fig. 2). Notably, the frequency of immunostained GAK-positive cells was positively correlated with the Gleason score (Fig. 2F, Table S1). Nuclear colocalization of GAK with its association partner AR [8] was observed in cancer cells from all of the GAK-positive surgical specimens (Fig. S1A). Unexpectedly, GAK did not phosphorylate AR *in vitro* (Fig. S1B). Co-administration of 60 µM luteolin and gefitinib to PC-3 cells had a greater effect on cell growth than administration of either compound alone (Fig. 5A). The increased activated/cleaved caspase-3 level (Fig. 5B) and subG1 (Fig. 5C) population detected by western blot and FACS analyses indicated that the growth inhibition was due to apoptosis. Erlotinib (Fig. S3A) and dietary luteolin supplements (Fig. S3C) showed similar inhibitory effects on PC-3 cell growth, suggesting that these compounds may also be useful as therapeutic agents at the bedside. It should be noted that the results obtained *in vivo* in the present study cannot be extended to androgen-dependent prostate cancers used in the IHC studies.

GAK is also overexpressed in KHOS and U2OS osteosarcoma cells; its overexpression is associated with poor prognosis, and siRNA-mediated knockdown of GAK decreases the proliferation of osteosarcoma cells [31], suggesting that GAK plays a pivotal role in the control of the proliferation rate of osteosarcoma cells. Unlike other osteosarcoma cell lines (HOS, MG-63, and KHOS/NP), U2OS cells lack expression of the EGFR, the well-known target of gefitinib; therefore, GAK is a putative *bona fide* target of gefitinib in these cells. Nonetheless, a recent study demonstrated similar effects of gefitinib on the viabilities of U2OS, HOS, MG-63, and KHOS/NP cells, although its effects were not conspicuous [32]. This finding adds extra credence to the theory that GAK is the inhibitory target of gefitinib in U2OS cells. Similar to PC-3 cells, co-administration of 60 µM luteolin and 20 µM gefitinib to U2OS cells had a greater effect on cell growth than administration of either compound alone (Fig. S5B). These findings may lead to the development of new therapeutic options for prostate cancer and osteosarcoma.

*In vitro* kinase assays using PP2A B′γ as a substrate [22] showed that gefitinib and erlotinib inhibit GAK with similar potency to SB203580, a well-known GAK inhibitor (Fig. 4A). Moreover, the flavonoid luteolin, but not the polyphenol resveratrol, also inhibited GAK activity (Fig. 4B). The cumulative inhibitory effects of luteolin and gefitinib on GAK activity suggest that these agents may bind to distinct sites of the GAK protein. Notably, the lack of GAK activity in GAK-kd MEFs caused constitutive hyper-phosphorylation and abnormal cytoplasmic localization of the EGFR (Fig. 3), which accelerated dephosphorylation of pERK1/2, leading to premature diminution of EGFR signaling. Therefore, lack of GAK activity disrupts EGFR-mediated signaling pathways. In addition, luteolin reduced the GAK protein level in a proteasome-independent manner (Fig. S4B-iii) and miRNA arrays demonstrated that miR-630 and miR-5703 were induced following gefitinib or luteolin treatment of PC-3 cells (Table S3), suggesting their involvement in the inhibition of GAK mRNA translation. Indeed, overexpression of miR-630 induced growth arrest of PC-3 cells (Fig. 6B). Similarly, recent studies showed that up-regulation of miR-630 induces apoptosis in pancreatic cancer cells [33] and cisplatin-induced miR-630 expression modulates the intrinsic apoptosis pathway in non-small cell lung cancer A549 cells [34].

**Figure 7:**
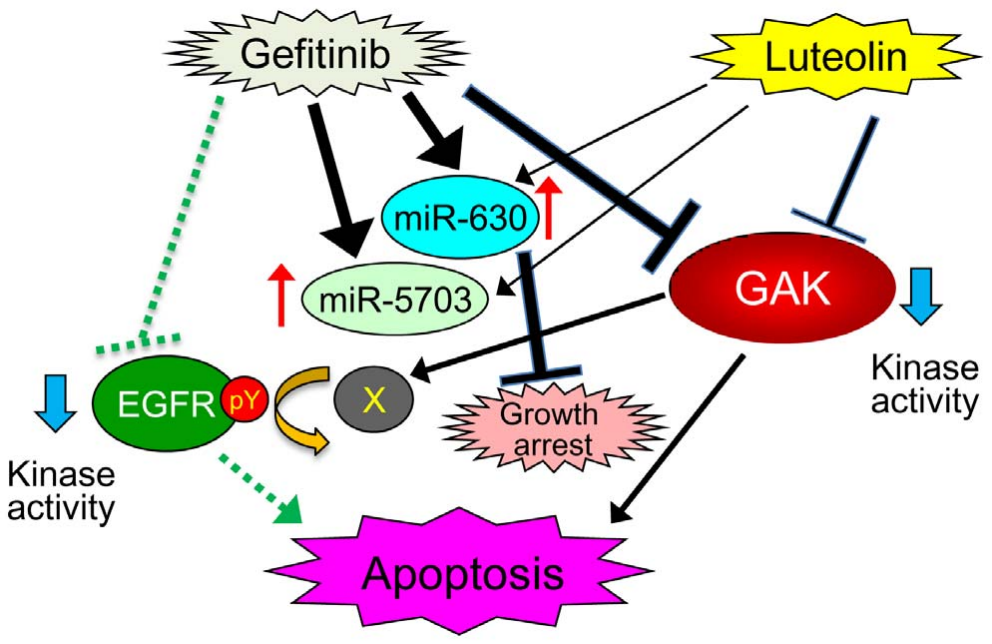
Working model showing the molecular mechanisms involved in the induction of growth arrest and apoptosis of PC-3 cells by luteolin and gefitinib. The arrows and T-shaped lines signify positive and negative actions, respectively. The thickness of each line is proportional to the strength of the denoted action. The dashed green line and arrow indicate that these actions were verified by another group [10]. The red and blue arrows indicate up-regulation and down-regulation, respectively. Down-regulation of GAK leads to hyper-phosphorylation of tyrosine residues (pY) in EGFR.

Based on the results described here, we propose a working model of the molecular mechanisms by which co-administration of gefitinib and luteolin induces efficient growth arrest and apoptosis of PC-3 cells (Fig. 7). Gefitinib and luteolin have cumulative inhibitory effects on the Ser/Thr kinase activity of GAK; their co-administration induces apoptosis as efficiently as inhibition of EGFR kinase activity. Down-regulation of GAK leads to hyper-phosphorylation of tyrosine residues (pY) in EGFR and alters the spectrum of downstream signaling. GAK appears to be essential for cell death because co-administration of gefitinib and luteolin induced efficient death of EGFR-deficient U2OS cells. Gefitinib also induces expression of miR-630 and miR-5703, leading to growth arrest of cells. GAK is the major therapeutic target of Parkinson's disease and novel chemicals that inhibit its kinase activity are currently being developed [35]; gefitinib and luteolin may also be clinically useful for the treatment of prostate cancer and osteosarcoma.

## Supporting Information

Figure S1 **GAK and AR are colocalized in nuclei of human prostate cancer cells** ***in vivo*** **.** (A–D) Representative images of radical prostatectomy sections immunostained with an anti-GAK (A, B) or anti-AR (C, D) antibody. Panels B and D show enlarged views of the boxed regions in panels A and C, respectively. Scale bars = 50 µm. (E) GAK does not phosphorylate the AR directly in vitro. SDS-PAGE analysis of proteins subjected to in vitro kinase assays using 32P-γATP. GST-tagged AR and PP2A B′γ (positive control) were used as substrates for phosphorylation by GST-tagged GAK. Aurora A kinase and Lats1/2 kinase (in the presence or absence of its activator Mob1A) were used as positive control enzymes, and a kinase-dead (KD) form of Aurora A kinase was used as a negative control for phosphorylation of the AR. The gel was stained with SimplyBlue SafeStain (upper panel) and the bands were detected by autoradiography (lower panel). The large arrows indicate autophosphorylated GAK and the small arrows indicate phosphorylated PP2A B′γ. The large arrowheads indicate autophosphorylated Lats1/2 and the small arrowheads indicate the phosphorylated AR. The open arrowheads indicate putative degradation products of the phosphorylated AR.
(TIF)

Figure S2 **GAK and the AR do not colocalize with Ki67 in prostate cancer surgical specimens.** Anti-GAK (A–C), anti-AR (D–F), and anti-Ki67 (G–I) immunostaining of radical prostatectomy sections from three independent prostate cancer patients. Scale bar = 50 µm.
(TIF)

Figure S3 **DNA sequence at the site of putative gefitinib-responsive mutations in the EGFR gene of PC-3 cells.** DNA sequences of the EGFR gene around the E746-A750 deletion (A) and around the mutations T790M (B), D761Y (C) and L858R (D) in non-small-cell lung cancer cells were determined in PC-3 cells. Normal DNA sequences of PC-3 cells are highlighted by red squares.
(TIF)

Figure S4 **The effects gefitinib, erlotinib, luteolin, and dietary luteolin supplements on PC-3 cells.** (A) The effects of erlotinib on growth inhibition of PC-3 cells are similar to those of gefitinib and luteolin. The numbers of viable PC-3 cells at the indicated times after exposure to gefitinib, erlotinib, luteolin, gefitinib plus luteolin, or erlotinib plus luteolin. The asterisk signifies that the actual concentration was slightly less than 100 µM because a trace amount of crystalized reagents were observed in the tissue culture medium. Data are represented as the mean ± SEM of n = 3 independent experiments for each condition.represented as the mean ± SEM of n = 3 independent experiments for each condition. (B) Resveratrol does not augment the inhibitory effects of luteolin and gefitinib on PC-3 cell growth. (i), the numbers of viable PC-3 cells 24, 48, and 72 h after the addition of solvent (DMSO), 60 µM resveratrol, 60 µM luteolin plus 60 µM gefitinib, or a combination of all three drugs. The data are represented as the mean ± SEM of n = 3 independent experiments at each concentration. (ii), FACS analysis of PC-3 cells 72 h after the addition of DMSO, 60 µM resveratrol, 60 µM luteolin plus 60 µM gefitinib, or a combination of all three drugs. (iii), the percentages of PC-3 cells in the indicated stages of the cell cycle after exposure to the same treatments for 72 h. (C) Dietary luteolin supplements cause growth inhibition of PC-3 cells. The numbers of viable PC-3 cells after exposure to dietary luteolin supplements (LutiMaxTM, CalComp Nutrition Inc.; and Oryza Oil & Fat Chemical Co., Ltd) for 72 h. Solvent alone (DMSO) was used as a control. The data are represented as the mean ± SEM of n = 3 independent experiments. (D) Co-administration of luteolin and gefitinib to PC-3 cells for 24 h induces very little cell death. (i), FACS analysis of PC-3 cells exposed to solvent alone (DMSO), 60 µM luteolin, 60 µM gefitinib, or a combination of luteolin and gefitinib for 24 h. (ii), the percentages of cells in the indicated phases of the cell cycle after exposure to the same treatments for 24 h.
(TIF)

Figure S5 **Lefty1 mRNA levels, but not protein levels, are affected by gefitinib and/or luteolin.** (A) Lefty1 mRNA expression is up-regulated in PC-3 cells exposed to gefitinib and/or luteolin for 24 h. Expression profiles of the genes whose mRNA levels were altered conspicuously after PC-3 cells were exposed to DMSO, gefitinib, luteolin, or gefitinib and luteolin for 24 h. Control measurements were taken prior to the exposures (0 hr_rep1 and 0 hr_rep2). Data represent the results of duplicate measurements. The expression profiles of Lefty1 and Lefty2 gene are indicated. The ordinate scale indicates relative log2 ratios. (B) Confirmation that Lefty1 mRNA levels, but not protein levels, are affected by gefitinib and/or luteolin. (i), duplicate qRT-PCR analyses of Lefty1 mRNA expression in untreated (0–1) cells and cells exposed to DMSO, gefitinib, luteolin, or gefitinib and luteolin for 24 h. The graphs show Lefty1 mRNA expression levels relative to those of 0 h (a.u., arbitrary unit). In each graph, the data are represented as the mean ± SEM of n = 3 replicates. (ii), immunoblot analyses of Lefty1, GAK and α-tubulin (control) protein levels in PC-3 cells exposed to DMSO, gefitinib, luteolin, or gefitinib and luteolin for 24, 48, or 72 h. The horizontal arrowhead indicates the bona fide Lefty1 protein band. The tilted arrowheads indicate down-regulation of GAK protein expression at 72 h. (iii), immunoblot analyses of Lefty1, GAK, and α-tubulin (control) protein levels in PC-3 cells treated with or without 60 µM luteolin, 50 µg/ml of the proteasome inhibitor MG132, and 0.5 µg/ml of the Lefty1-specific siRNAs (Y and Z). Identification of bona fide Lefty1 band (arrowhead) in western blot was performed by siRNA-mediated knockdown after addition of luteolin; siRNAs were added 24 h before addition of luteolin. MG132 was added 22 h after addition of luteolin; then cells were incubated for additional 2 h before RNA extraction. The arrow indicates the appearance of a smaller (faster migrating) GAK band after the addition of luteolin.
(TIF)

Figure S6 **Co-administration of luteolin and gefitinib causes growth inhibition of U2OS osteosarcoma cells.** (A) The numbers of viable U2OS cells 24, 48, and 72 h after the addition of solvent alone (DMSO), 60 µM luteolin, 60 µM gefitinib, or 60 µM luteolin plus 60 µM gefitinib. (B) The numbers of viable U2OS cells 24, 48, and 72 h after the addition of solvent alone (DMSO), 60 µM luteolin, 20 µM gefitinib, or 60 µM luteolin plus 20 µM gefitinib. A, B, the data are represented as the mean ± SEM of n = 3 independent experiments for each condition.
(TIF)

Table S1 **The ages, Gleason scores, and GAK levels of the 42 prostate cancer patients enrolled in the study.** The shaded rows indicate patients with high GAK expression levels.
(TIF)

Table S2 **List of the genes that were up-regulated conspicuously following exposure of PC-3 cells to 60 µM gefitinib, 60 µM luteolin, or a combination of these drugs (Lut+Gef) for 24 h.** The fold-changes in gene expression were calculated relative to those in untreated cells.
(TIF)

Table S3 **The miRNAs that were up-regulated in PC-3 cells treated with 60 µM luteolin and/or 60 µM gefitinib for 24 h.** Columns 2–4 show fold-changes in expression relative to DMSO-treated PC-3 cells. Columns 5–8 show the raw signal intensities. Notable miRNAs (miR-630 and miR-5703) are highlighted.
(TIF)
