# Supplementary figures and images for: Gefitinib and Luteolin Cause Growth Arrest of Human Prostate Cancer PC-3 Cells via Inhibition of Cyclin G-Associated Kinase and Induction of miR-630
Source: PLoS One. 2014 Jun 27;9(6):e100124. doi: 10.1371/journal.pone.0100124 (PMC4074034; doi:10.1371/journal.pone.0100124)

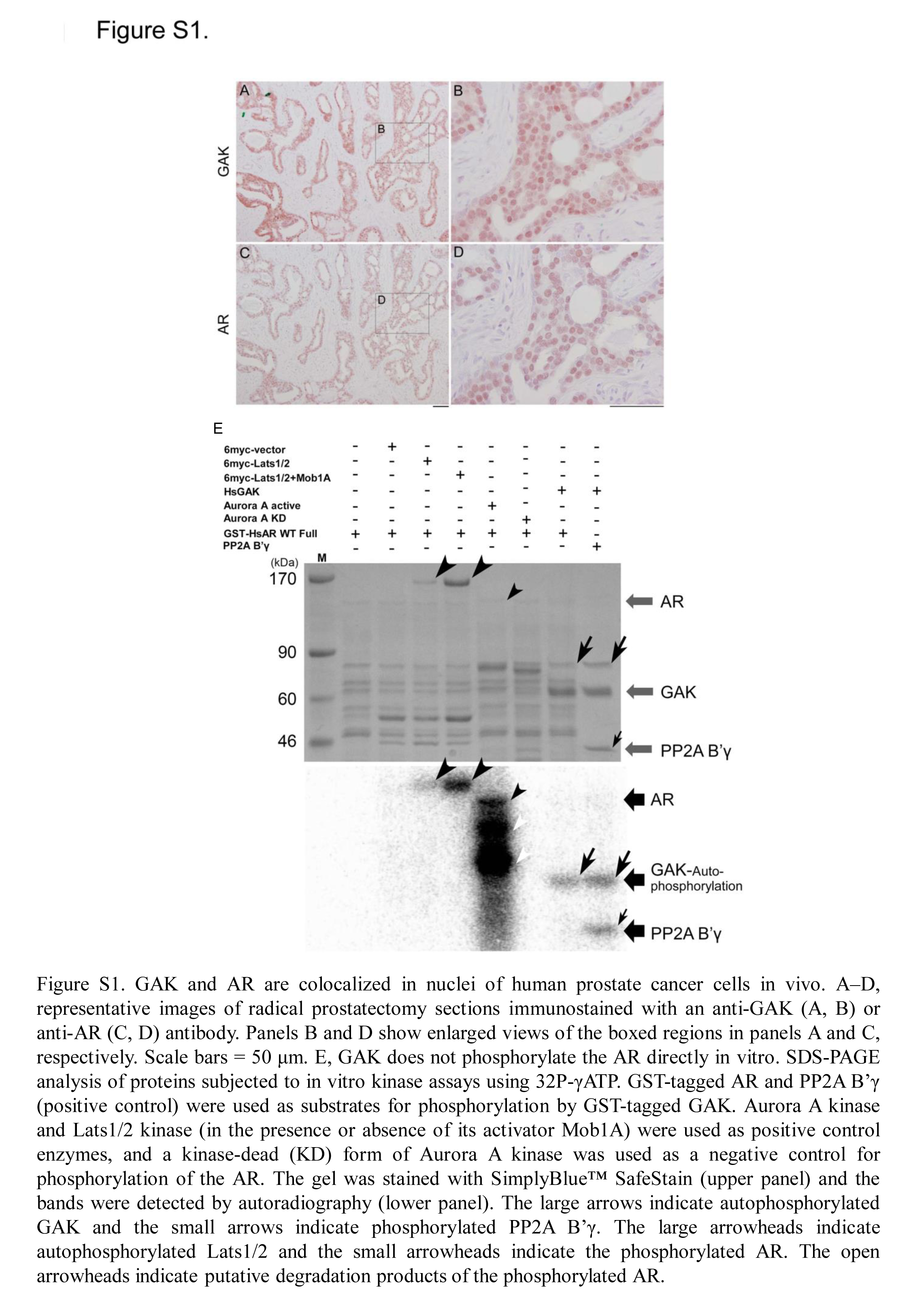

Supplement: Figure S1 — GAK and AR are colocalized in nuclei of human prostate cancer cells in vivo . (A–D) Representative images of radical prostatectomy sections immunostained with an anti-GAK (A, B) or anti-AR (C, D) antibody. Panels B and D show enlarged views of the boxed regions in panels A and C, respectively. Scale bars = 50 µm. (E) GAK does not phosphorylate the AR directly in vitro. SDS-PAGE analysis of proteins subjected to in vitro kinase assays using 32P-γATP. GST-tagged AR and PP2A B′γ (positive control) were used as substrates for phosphorylation by GST-tagged GAK. Aurora A kinase and Lats1/2 kinase (in the presence or absence of its activator Mob1A) were used as positive control enzymes, and a kinase-dead (KD) form of Aurora A kinase was used as a negative control for phosphorylation of the AR. The gel was stained with SimplyBlue SafeStain (upper panel) and the bands were detected by autoradiography (lower panel). The large arrows indicate autophosphorylated GAK and the small arrows indicate phosphorylated PP2A B′γ. The large arrowheads indicate autophosphorylated Lats1/2 and the small arrowheads indicate the phosphorylated AR. The open arrowheads indicate putative degradation products of the phosphorylated AR. (TIF) [file pone.0100124.s001.tif]

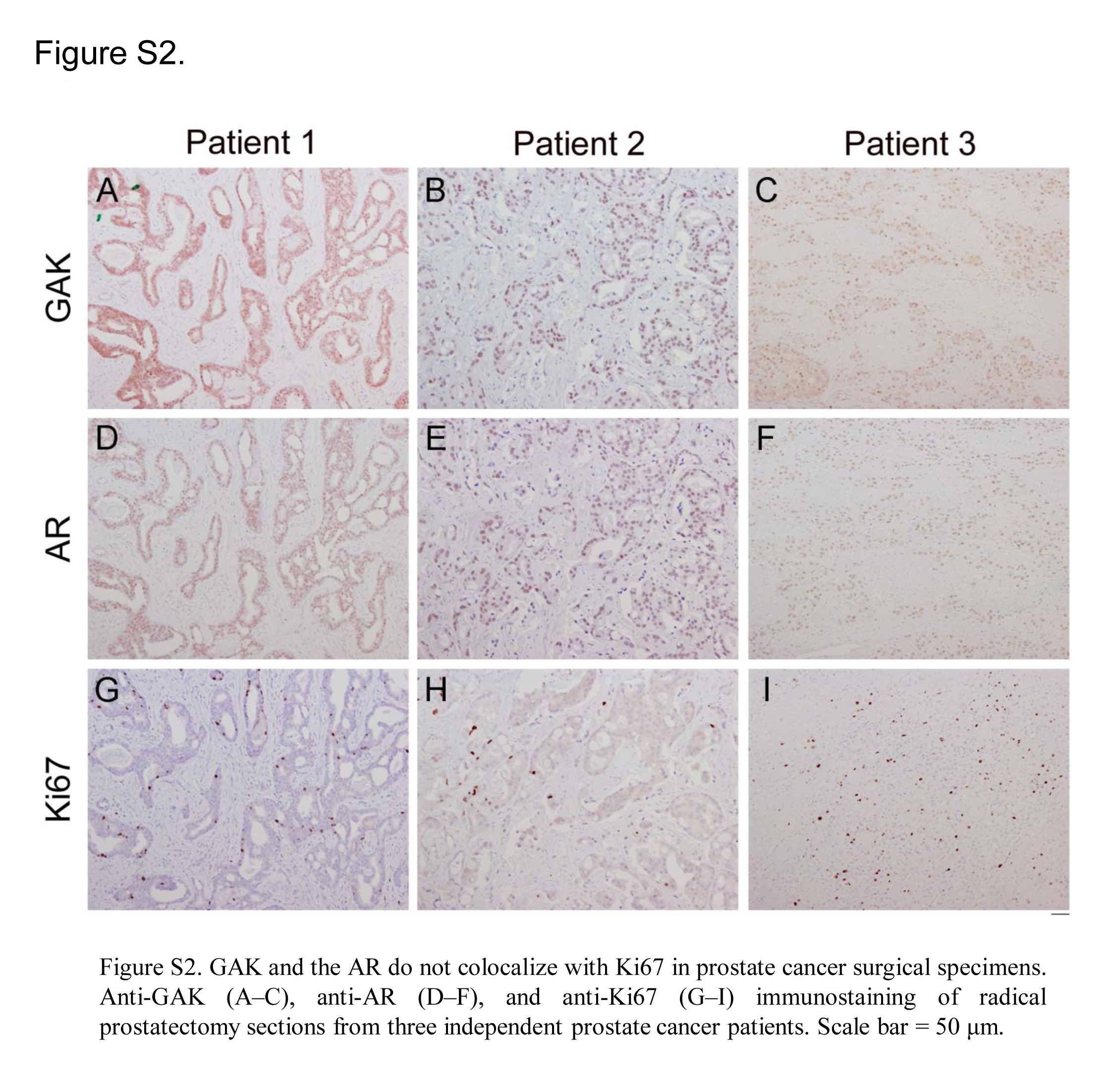

Supplement: Figure S2 — GAK and the AR do not colocalize with Ki67 in prostate cancer surgical specimens. Anti-GAK (A–C), anti-AR (D–F), and anti-Ki67 (G–I) immunostaining of radical prostatectomy sections from three independent prostate cancer patients. Scale bar = 50 µm. (TIF) [file pone.0100124.s002.tif]

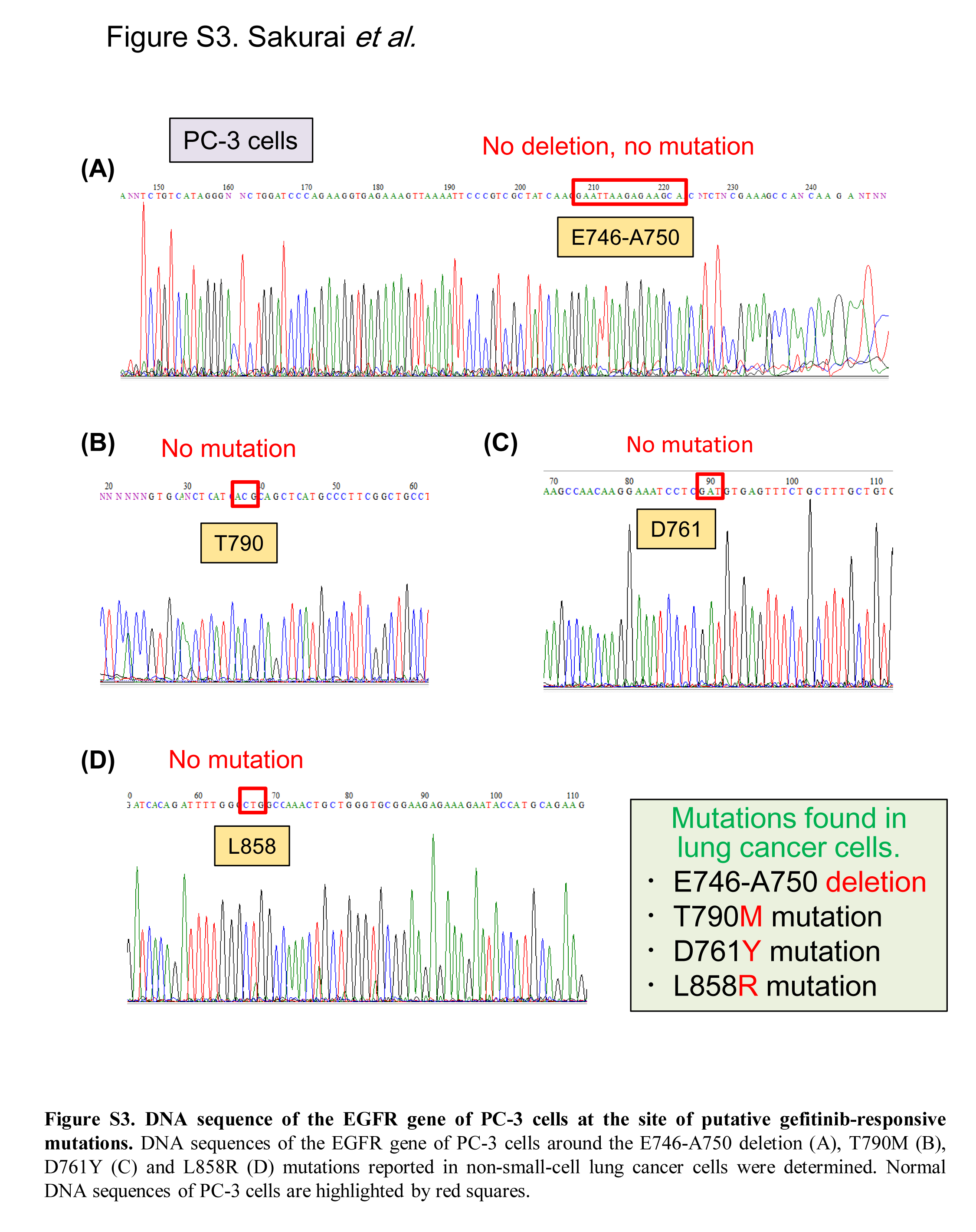

Supplement: Figure S3 — DNA sequence at the site of putative gefitinib-responsive mutations in the EGFR gene of PC-3 cells. DNA sequences of the EGFR gene around the E746-A750 deletion (A) and around the mutations T790M (B), D761Y (C) and L858R (D) in non-small-cell lung cancer cells were determined in PC-3 cells. Normal DNA sequences of PC-3 cells are highlighted by red squares. (TIF) [file pone.0100124.s003.tif]

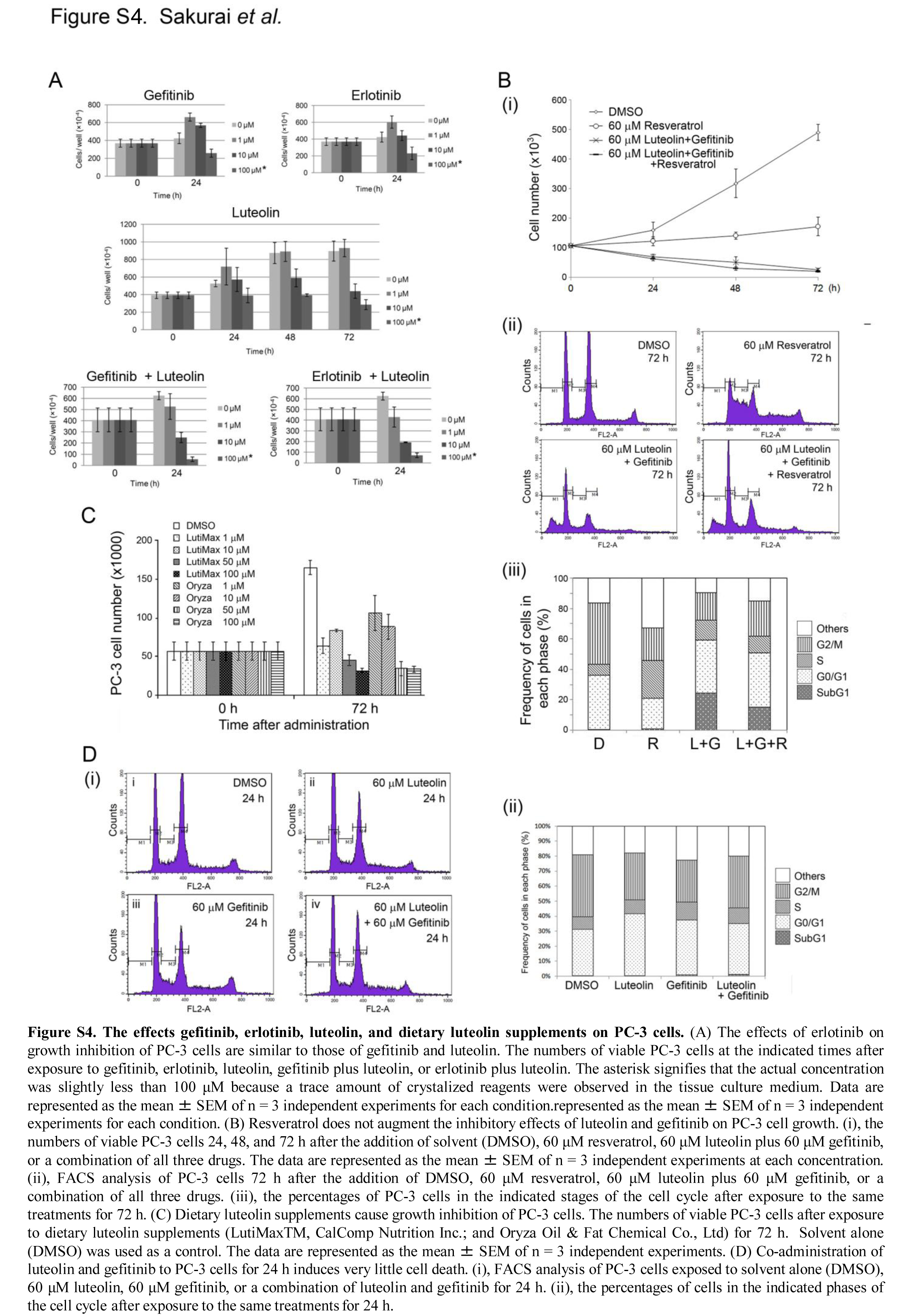

Supplement: Figure S4 — The effects gefitinib, erlotinib, luteolin, and dietary luteolin supplements on PC-3 cells. (A) The effects of erlotinib on growth inhibition of PC-3 cells are similar to those of gefitinib and luteolin. The numbers of viable PC-3 cells at the indicated times after exposure to gefitinib, erlotinib, luteolin, gefitinib plus luteolin, or erlotinib plus luteolin. The asterisk signifies that the actual concentration was slightly less than 100 µM because a trace amount of crystalized reagents were observed in the tissue culture medium. Data are represented as the mean ± SEM of n = 3 independent experiments for each condition.represented as the mean ± SEM of n = 3 independent experiments for each condition. (B) Resveratrol does not augment the inhibitory effects of luteolin and gefitinib on PC-3 cell growth. (i), the numbers of viable PC-3 cells 24, 48, and 72 h after the addition of solvent (DMSO), 60 µM resveratrol, 60 µM luteolin plus 60 µM gefitinib, or a combination of all three drugs. The data are represented as the mean ± SEM of n = 3 independent experiments at each concentration. (ii), FACS analysis of PC-3 cells 72 h after the addition of DMSO, 60 µM resveratrol, 60 µM luteolin plus 60 µM gefitinib, or a combination of all three drugs. (iii), the percentages of PC-3 cells in the indicated stages of the cell cycle after exposure to the same treatments for 72 h. (C) Dietary luteolin supplements cause growth inhibition of PC-3 cells. The numbers of viable PC-3 cells after exposure to dietary luteolin supplements (LutiMaxTM, CalComp Nutrition Inc.; and Oryza Oil & Fat Chemical Co., Ltd) for 72 h. Solvent alone (DMSO) was used as a control. The data are represented as the mean ± SEM of n = 3 independent experiments. (D) Co-administration of luteolin and gefitinib to PC-3 cells for 24 h induces very little cell death. (i), FACS analysis of PC-3 cells exposed to solvent alone (DMSO), 60 µM luteolin, 60 µM gefitinib, or a combination of luteolin and gefitinib for 24 h. ( [file pone.0100124.s004.tif]

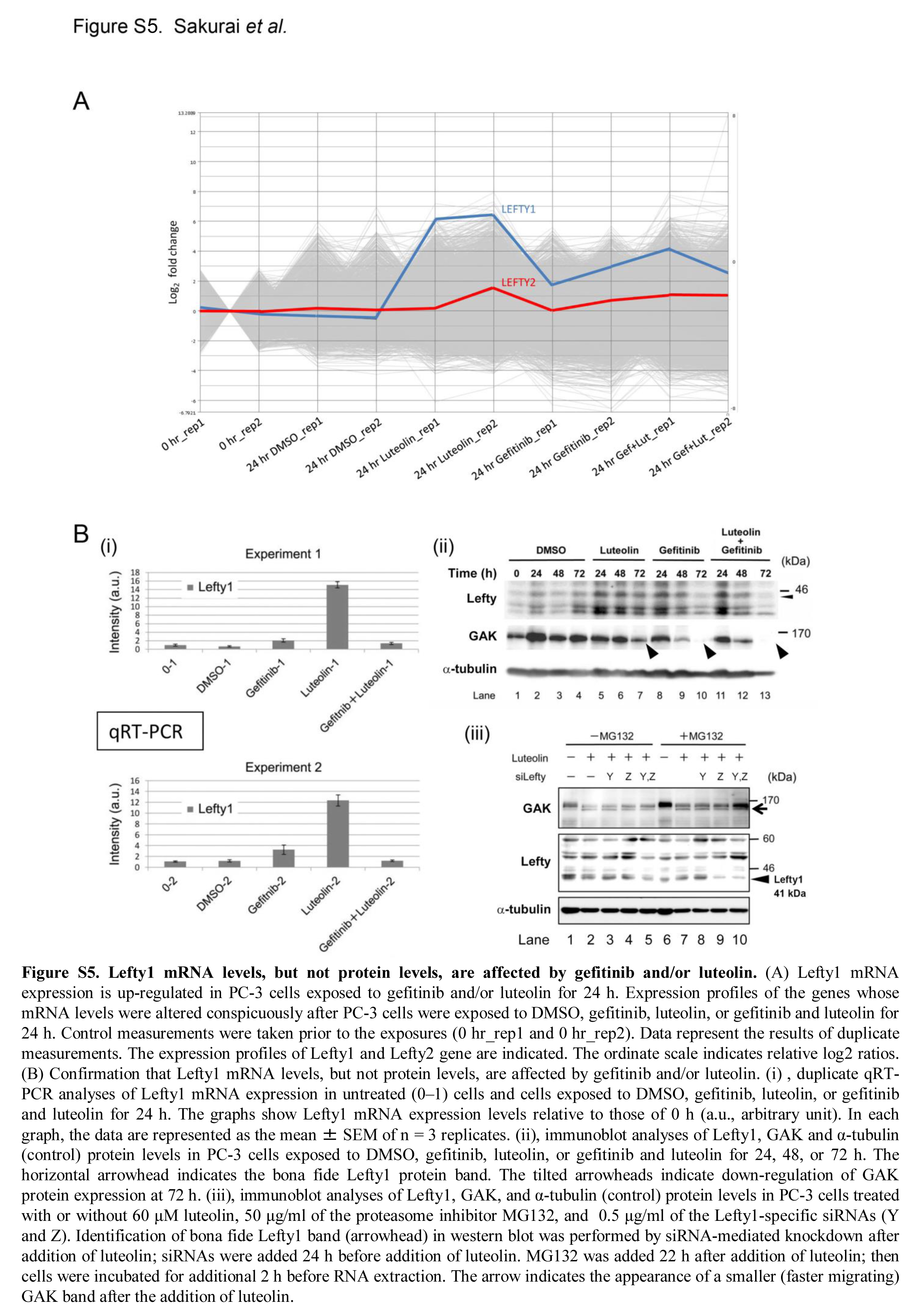

Supplement: Figure S5 — Lefty1 mRNA levels, but not protein levels, are affected by gefitinib and/or luteolin. (A) Lefty1 mRNA expression is up-regulated in PC-3 cells exposed to gefitinib and/or luteolin for 24 h. Expression profiles of the genes whose mRNA levels were altered conspicuously after PC-3 cells were exposed to DMSO, gefitinib, luteolin, or gefitinib and luteolin for 24 h. Control measurements were taken prior to the exposures (0 hr_rep1 and 0 hr_rep2). Data represent the results of duplicate measurements. The expression profiles of Lefty1 and Lefty2 gene are indicated. The ordinate scale indicates relative log2 ratios. (B) Confirmation that Lefty1 mRNA levels, but not protein levels, are affected by gefitinib and/or luteolin. (i), duplicate qRT-PCR analyses of Lefty1 mRNA expression in untreated (0–1) cells and cells exposed to DMSO, gefitinib, luteolin, or gefitinib and luteolin for 24 h. The graphs show Lefty1 mRNA expression levels relative to those of 0 h (a.u., arbitrary unit). In each graph, the data are represented as the mean ± SEM of n = 3 replicates. (ii), immunoblot analyses of Lefty1, GAK and α-tubulin (control) protein levels in PC-3 cells exposed to DMSO, gefitinib, luteolin, or gefitinib and luteolin for 24, 48, or 72 h. The horizontal arrowhead indicates the bona fide Lefty1 protein band. The tilted arrowheads indicate down-regulation of GAK protein expression at 72 h. (iii), immunoblot analyses of Lefty1, GAK, and α-tubulin (control) protein levels in PC-3 cells treated with or without 60 µM luteolin, 50 µg/ml of the proteasome inhibitor MG132, and 0.5 µg/ml of the Lefty1-specific siRNAs (Y and Z). Identification of bona fide Lefty1 band (arrowhead) in western blot was performed by siRNA-mediated knockdown after addition of luteolin; siRNAs were added 24 h before addition of luteolin. MG132 was added 22 h after addition of luteolin; then cells were incubated for additional 2 h before RNA extraction. The arrow indicates the appearance of a smaller (faster migr [file pone.0100124.s005.tif]

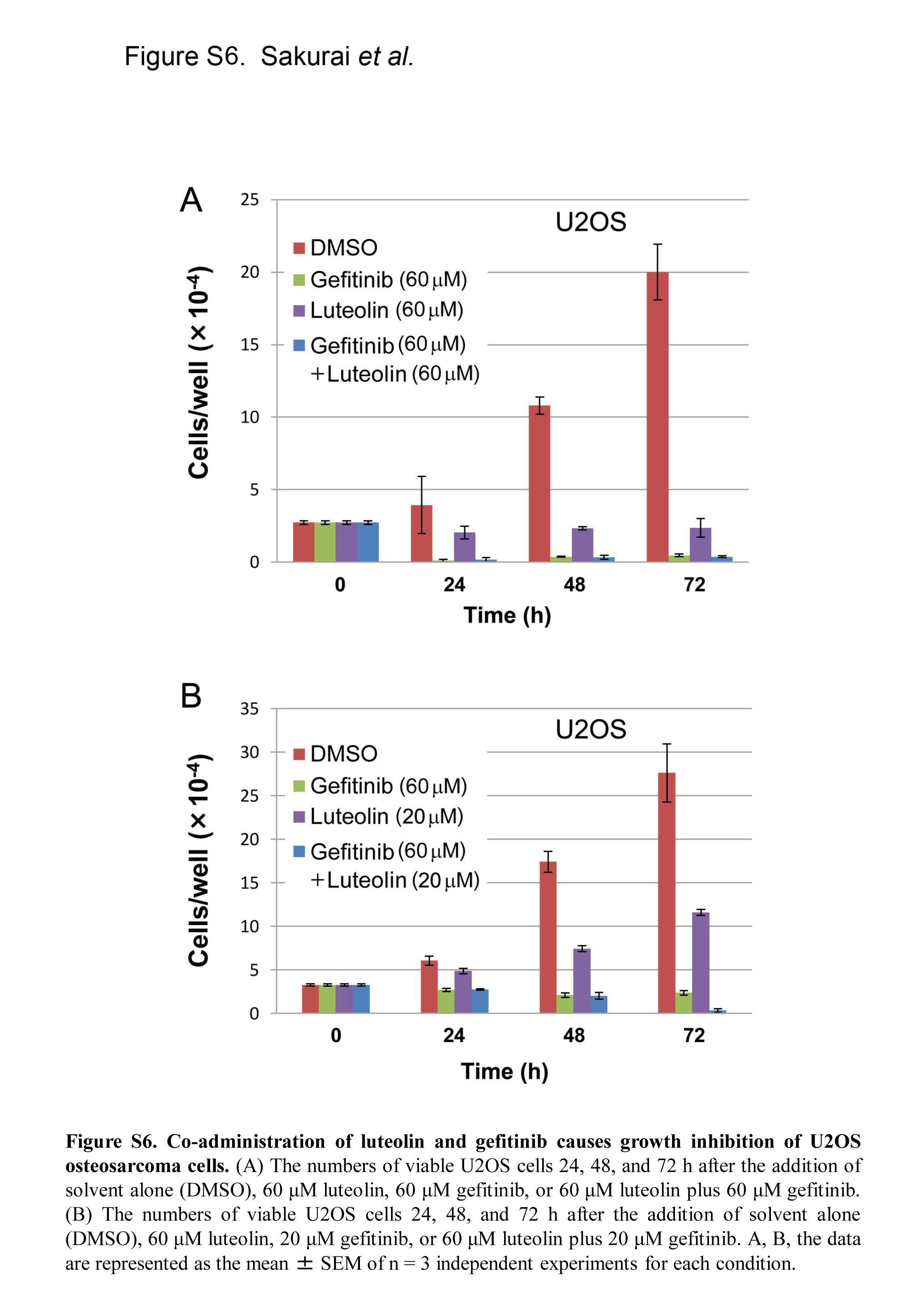

Supplement: Figure S6 — Co-administration of luteolin and gefitinib causes growth inhibition of U2OS osteosarcoma cells. (A) The numbers of viable U2OS cells 24, 48, and 72 h after the addition of solvent alone (DMSO), 60 µM luteolin, 60 µM gefitinib, or 60 µM luteolin plus 60 µM gefitinib. (B) The numbers of viable U2OS cells 24, 48, and 72 h after the addition of solvent alone (DMSO), 60 µM luteolin, 20 µM gefitinib, or 60 µM luteolin plus 20 µM gefitinib. A, B, the data are represented as the mean ± SEM of n = 3 independent experiments for each condition. (TIF) [file pone.0100124.s006.tif]

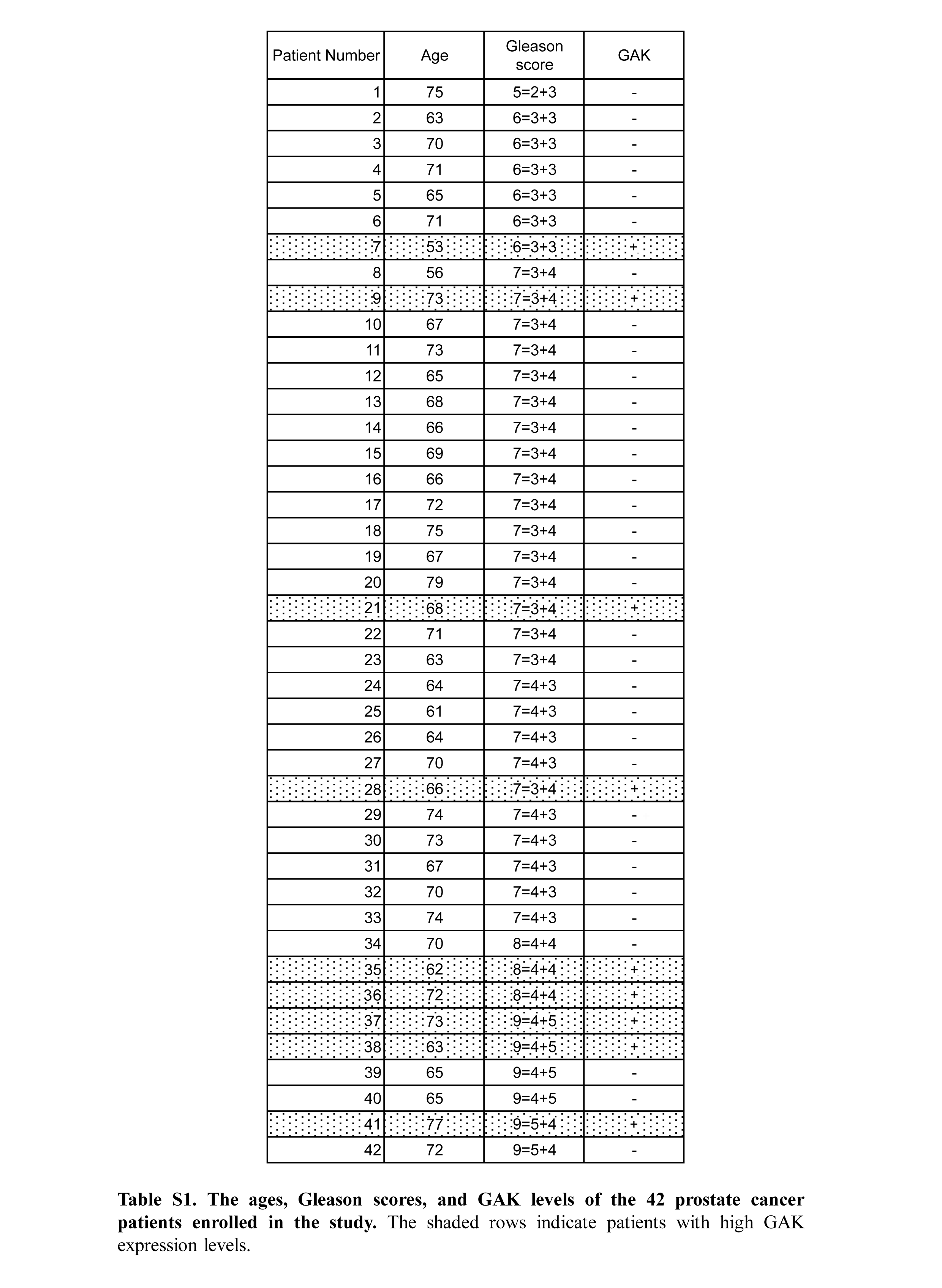

Supplement: Table S1 — The ages, Gleason scores, and GAK levels of the 42 prostate cancer patients enrolled in the study. The shaded rows indicate patients with high GAK expression levels. (TIF) [file pone.0100124.s007.tif]

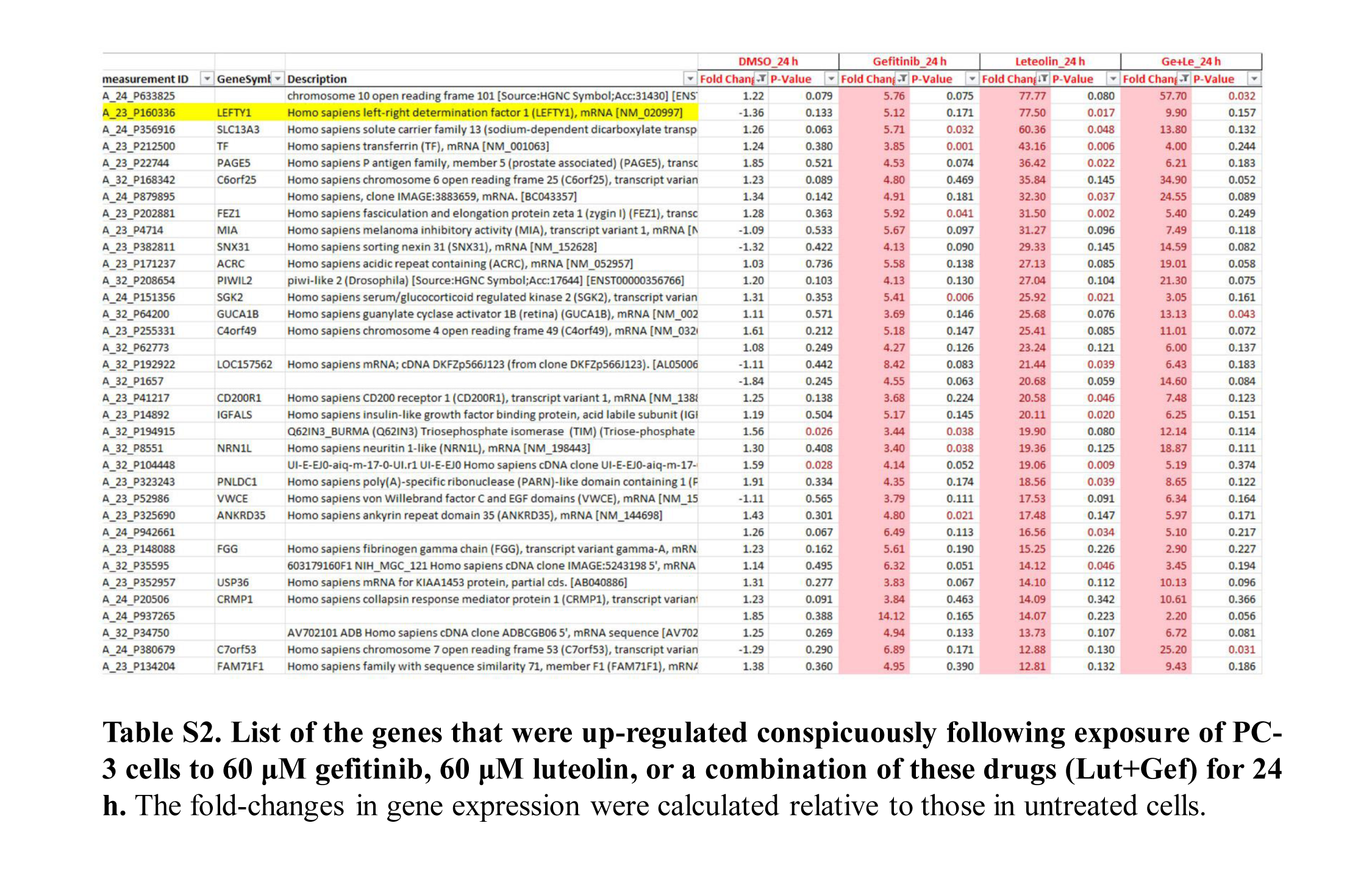

Supplement: Table S2 — List of the genes that were up-regulated conspicuously following exposure of PC-3 cells to 60 µM gefitinib, 60 µM luteolin, or a combination of these drugs (Lut+Gef) for 24 h. The fold-changes in gene expression were calculated relative to those in untreated cells. (TIF) [file pone.0100124.s008.tif]

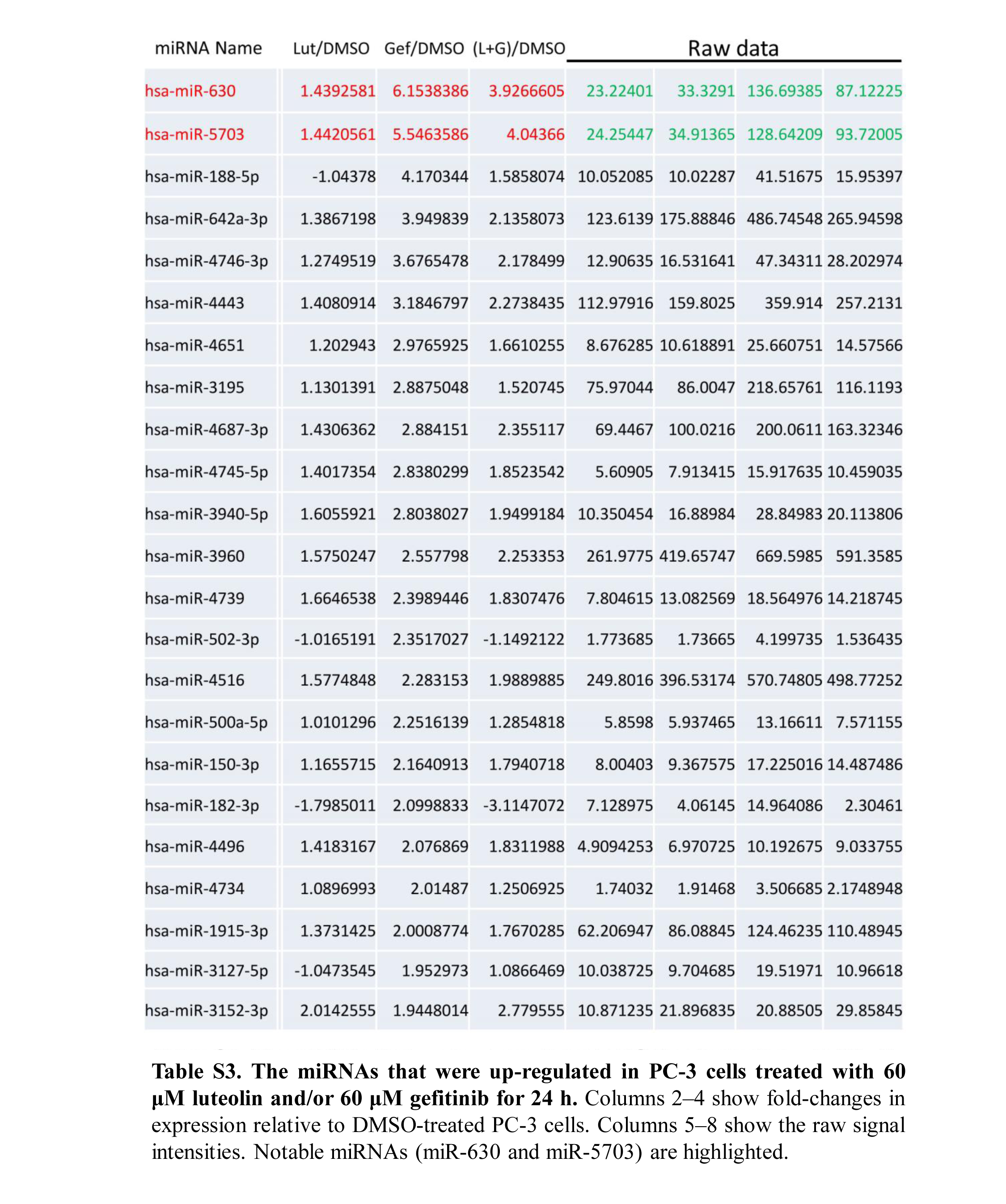

Supplement: Table S3 — The miRNAs that were up-regulated in PC-3 cells treated with 60 µM luteolin and/or 60 µM gefitinib for 24 h. Columns 2–4 show fold-changes in expression relative to DMSO-treated PC-3 cells. Columns 5–8 show the raw signal intensities. Notable miRNAs (miR-630 and miR-5703) are highlighted. (TIF) [file pone.0100124.s009.tif]
